# Regional Activation of Myosin II in Cancer Cells Drives Tumor Progression via a Secretory Cross-Talk with the Immune Microenvironment

**DOI:** 10.1016/j.cell.2018.12.038

**Published:** 2019-02-07

**Authors:** Mirella Georgouli, Cecilia Herraiz, Eva Crosas-Molist, Bruce Fanshawe, Oscar Maiques, Anna Perdrix, Pahini Pandya, Irene Rodriguez-Hernandez, Kristina M. Ilieva, Gaia Cantelli, Panagiotis Karagiannis, Silvia Mele, Hoyin Lam, Debra H. Josephs, Xavier Matias-Guiu, Rosa M. Marti, Frank O. Nestle, Jose L. Orgaz, Ilaria Malanchi, Gilbert O. Fruhwirth, Sophia N. Karagiannis, Victoria Sanz-Moreno

**Affiliations:** 1 Barts Cancer Institute, John Vane Science Building, Charterhouse Square, Queen Mary University of London, London EC1M 6BQ, UK; 2 Randall Centre for Cell and Molecular Biophysics, New Hunt’s House, Guy’s Campus, King’s College London, London SE1 1UL, UK; 3 Department of Imaging Chemistry and Biology, Division of Imaging Sciences and Biomedical Engineering, St. Thomas Hospital, King’s College London, London SE1 7EH, UK; 4 Tumour-Stroma Interactions in Cancer Laboratory, The Francis Crick Institute, 1 Midland Road, London NW1 1AT, UK; 5 St John’s Institute of Dermatology, King’s College London and National Institute for Health Research Biomedical Research Centre at Guy’s and St Thomas’ Hospitals and King’s College London, London SE1 9RT, UK; 6 Department of Oncology, Haematology and Stem Cell Transplantation, University Hospital of Hamburg Eppendorf, Hamburg 20246, Germany; 7 Departments of Pathology Hospital U Arnau de Vilanova and Hospital U de Bellvitge, IRBLLEIDA, IDIBELL, University of Lleida, CIBERONC, Lleida, Spain; 8 Department of Dermatology, Hospital U Arnau de Vilanova, IRBLLEIDA, University of Lleida, CIBERONC, Lleida, Spain; 9 School of Cancer and Pharmaceutical Sciences, Guy’s Hospital, King’s College London, London SE1 9RT, UK

**Keywords:** ROCK-Myosin II, rounded-amoeboid melanoma cells, tumor invasive front, protein secretion, NF-κB, macrophages

## Abstract

ROCK-Myosin II drives fast rounded-amoeboid migration in cancer cells during metastatic dissemination. Analysis of human melanoma biopsies revealed that amoeboid melanoma cells with high Myosin II activity are predominant in the invasive fronts of primary tumors in proximity to CD206^+^CD163^+^ tumor-associated macrophages and vessels. Proteomic analysis shows that ROCK-Myosin II activity in amoeboid cancer cells controls an immunomodulatory secretome, enabling the recruitment of monocytes and their differentiation into tumor-promoting macrophages. Both amoeboid cancer cells and their associated macrophages support an abnormal vasculature, which ultimately facilitates tumor progression. Mechanistically, amoeboid cancer cells perpetuate their behavior via ROCK-Myosin II-driven IL-1α secretion and NF-κB activation. Using an array of tumor models, we show that high Myosin II activity in tumor cells reprograms the innate immune microenvironment to support tumor growth. We describe an unexpected role for Myosin II dynamics in cancer cells controlling myeloid function via secreted factors.

## Highlights

• Invasive tumor fronts are enriched in rounded-amoeboid cancer cells high in Myosin II
• Myosin II activity in these cells regulates an immunomodulatory secretome
• The secreted cytokines and chemokines can induce tumor-promoting macrophages
• ROCK-Myosin II and IL-1α/NF-κB cross-talk supports this secretory phenotype

## Graphical Abstract

**Figure:**
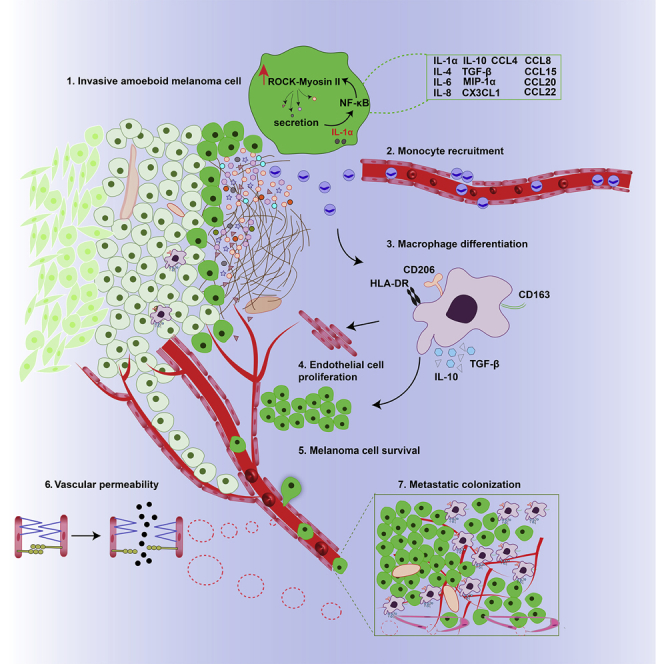

## Teaser

Myosin II activation at the tumor edge promotes invasion and also a secretory phenotype that reshapes the local environment and vasculature to support tumor growth.

## Introduction

Metastasis accounts for >90% of cancer-related deaths, indicating an urgent need for clinical management (Friedl and Wolf, 2003). To leave the primary tumor, cancer cells disseminate using different migration modes (Pandya et al., 2017). Although collective cell migration is important for tissue remodeling, single-cell migration (rounded-amoeboid or elongated-mesenchymal) allows transport, both locally and to distant sites along with invasion through basement membranes (Giampieri et al., 2009). Actomyosin contractility driven by Myosin II controls cytoskeletal remodeling and tumor dissemination (Rodriguez-Hernandez et al., 2016). ROCK can directly phosphorylate myosin light chain 2 (MLC2) or indirectly decrease Myosin phosphatase (MYPT) activity increasing MLC2 phosphorylation (Ito et al., 2004). ROCK can also activate LIMK, which phosphorylates and inactivates cofilin resulting in F-actin stabilization (Yang et al., 1998). High levels of actomyosin contractility driven by Myosin II are key to sustain amoeboid bleb-based migration (Orgaz et al., 2014, Sahai and Marshall, 2003, Sanz-Moreno et al., 2011). Intravital imaging in melanoma and breast cancer mouse xenografts revealed amoeboid migration is favored in the tumor invasive fronts (IFs) (Herraiz et al., 2015, Sanz-Moreno et al., 2008, Sanz-Moreno et al., 2011, Tozluoğlu et al., 2013).

On the other hand, cancer-associated inflammation promotes tumorigenesis at many levels. Inflammation is enabled by the secretion of multiple factors and the recruitment of immune cells, like monocytes (Coussens and Werb, 2002). Monocytes differentiate to macrophages, which can change their phenotypes responding to microenvironmental signals. Classically activated macrophages are induced in response to pro-inflammatory stimuli, such as lipopolysaccharides (LPS) or interferon gamma (IFN-γ) and exhibit cytotoxic functions. Cytokines such as interleukin (IL)-4, IL-13, and IL-10, transforming growth factor beta (TGF-β), and/or glucocorticoids can support alternatively activated macrophages (AAMs) that promote tissue repair and tumor progression (Gordon, 2003).

Here, we investigated how Myosin II activity in cancer cells controls the secretion of factors regulating the tumor microenvironment (TME) via the establishment of a cross-talk with pro-inflammatory nuclear factor κB (NF-κB).

## Results

### Invasive Fronts of Human Melanomas Are Enriched in Amoeboid Melanoma Cells Close to Macrophages and Blood Vessels

**Figure 1:**
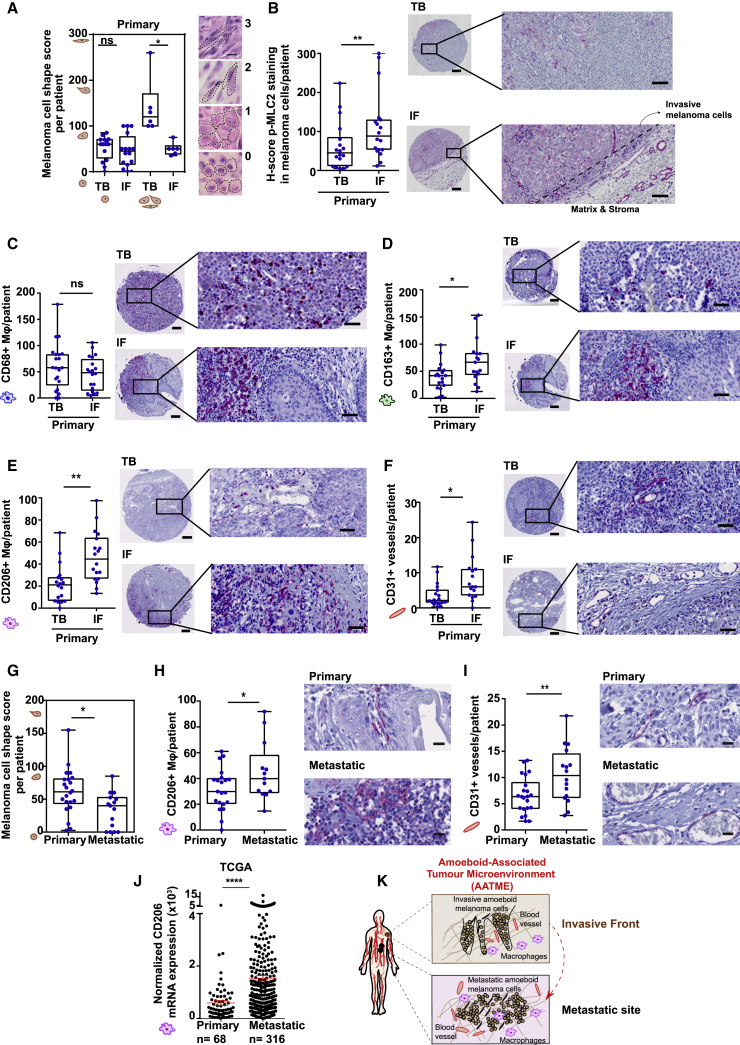
Invasive Fronts of Human Melanomas Are Enriched in Amoeboid Melanoma Cells Close to Macrophages and Blood Vessels (A) Melanoma cell-shape score in tumour body (TB) or invasive front (IF) of matched samples from human primary melanoma. Values range from 0 (all cells round) to 300 (all cells spindle). (B) H-score of p-MLC2 staining from patients in (A). Values range from 0 (no staining) to 400 (very intense staining). (C–F) (Left) Quantification and (right) representative images of (C) CD68^+^, (D) CD163^+^, (E) CD206^+^, and (F) CD31^+^ cells in TB and IF of primary melanomas. (G) Melanoma cell-shape score in primary and metastatic melanomas. (H and I) (Left) Quantification and (right) representative images of (H) CD206^+^ and (I) CD31^+^ cells in primary and metastatic melanomas. (J) mRNA levels of CD206 in primary (n = 68) and metastatic (n = 316) melanomas. Raw data were obtained from TCGA. (K) Schematic: IF and metastatic site of human melanoma. In (A)–(I), n = 24 primary and n = 16 metastatic melanomas. Scale bars, 200 μm for the tumor cores, 50 μm for all the focused images except (A) for the focused images showing the score where scale bar is 5 μm. All data are presented per patient. Average has been taken from 4 tumor cores per TB and 4 tumor cores per IF. In (A)–(F), matched TB and IF from same patients are presented. In (A)–(I), boxplots show min to max values. In (J), dot blot shows mean ± SEM. In (A)–(F), Wilcoxon matched-pairs signed-rank test is shown. In (G)–(J), t test is shown. ^∗^p < 0.05, ^∗∗^p < 0.01, ^∗∗∗^p < 0.001, ^∗∗∗∗^p < 0.0001. See also Figure S1 and Tables S3 and S4.

Using intravital imaging in xenograft melanoma models, we have previously reported an enrichment in amoeboid migration in the invasive edge of tumors (Herraiz et al., 2015, Sanz-Moreno et al., 2011). To test whether this was recapitulated in human patient tissues, we evaluated human melanoma biopsies using a tissue microarray (40 human melanoma lesions, cohort A) and a smaller cohort (7 melanoma lesions, cohort B). For both cohorts, matched tumor body (TB) and IF were included. IFs of human primary melanomas were found enriched in rounded cancer cells independently of the morphology in the TB (Figure 1A). Importantly, we found a regional increase in phosphorylated MLC2 (p-MLC2) levels in the IF (Figure 1B) indicative of high Myosin II activity. Our data suggest that the combination of roundness and increased p-MLC2 can define the amoeboid contractile cancer phenotype accurately in patients’ biopsies.

**Figure S1:**
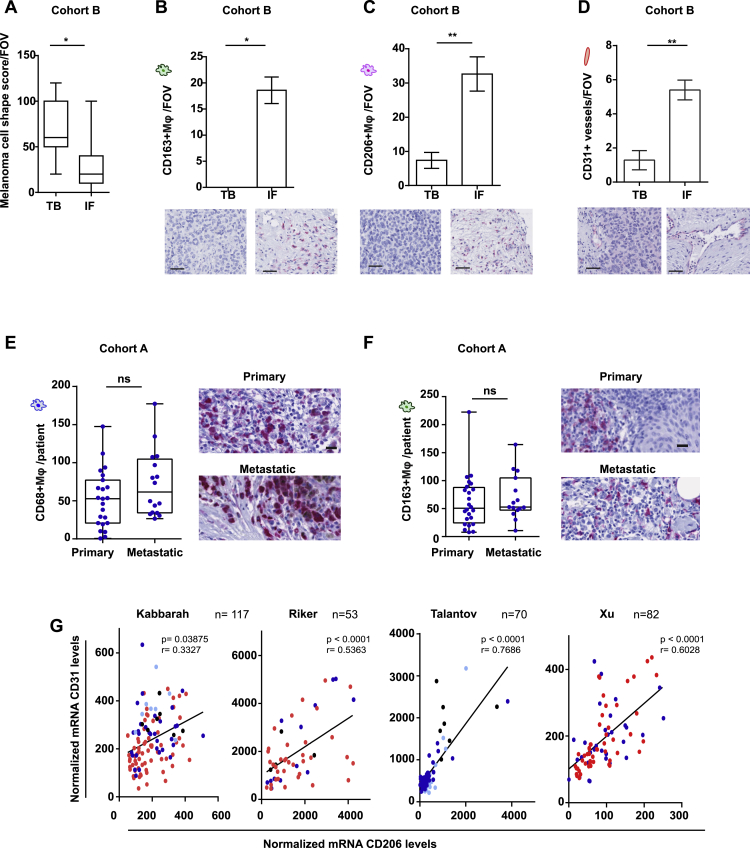
Invasive Fronts of Human Melanomas Are Enriched in Amoeboid Melanoma Cells with High Myosin II Activity in the Vicinity of Macrophages and Blood Vessels, Related to Figure 1 (A) Melanoma cell shape score in tumor body (TB) or invasive front (IF) of human melanoma biopsies in Cohort B, values ranging from 0 (all cells round) to 300 (all cells spindle) (See also STAR methods) (n = 7). (B–D) Average number (top) and representative images (below) of (B) CD163+ macrophages, (C) CD206+ macrophages and (D) CD31+ vessels, per field of view (FOV), in TB or IF in Cohort B. Scale bar, 50 μm (n = 4 for CD163, n = 5 for CD206, n = 6 for vessels). (E) Average number of CD68+ macrophages and (F) CD163+ macrophages, in primary and metastatic melanoma lesions in Cohort A. Data are presented per patient. (G) Scatterplot for correlation of CD31 and CD206 mRNA levels in normal skin (black), nevi (cyan), primary melanoma (blue), metastatic melanoma (red). Pearson’s r. Raw data obtained from the publicly available database GEO. (A, E, and F) Boxplots show min to max values. (B–D) Graphs show mean ± SEM. (A–D) Paired t test. (E and F) t test. ns p > 0.05,^∗^p < 0.05,^∗∗^p < 0.01,^∗∗∗^p < 0.001.

Interestingly, in both of our patient tissue cohorts, macrophages were a prominent population, in accordance with previous studies reporting that macrophages constitute up to 30% of the immune infiltrate in melanoma (Bröcker et al., 1988, Hussein, 2006). Furthermore, high tumor-associated macrophage (TAM) infiltration has been associated to poor prognosis (Zhang et al., 2015). We thus sought to identify regional differences in macrophage composition in patient cohort A. CD68 is a pan-macrophage marker, while CD163 and CD206 (Kakizaki et al., 2015) typify AAMs (Vogel et al., 2014). We found no regional difference in CD68^+^ cells (Figure 1C). However, both CD163^+^ and CD206^+^ TAMs were enriched in the IFs of tumors (Figures 1D and 1E). TAMs support angiogenesis (Chen et al., 2011), while they are located in perivascular tumor areas (Wyckoff et al., 2007). We observed elevated vessel density in the same tumor regions where macrophages were abundant (Figure 1F). Similar results were observed in cohort B (Figures S1A–S1D).

The TME in aggressive tumors may favor the formation of pre-metastatic “invasive niches” composed of cancer cells, endothelial cells, and macrophages (Joyce and Pollard, 2009). Indeed, metastatic melanoma lesions were enriched in both rounded melanoma cells (Figure 1G) and CD206^+^ TAMs (Figures 1H, S1E, and S1F) in proximity to blood vessels (Figure 1I). Using the Cancer Genome Atlas (TCGA) database, we found increased CD206 mRNA levels in metastatic versus primary human melanomas (n = 384) (Figure 1J). Furthermore, using Gene Expression Omnibus (GEO) database, we found a positive correlation between CD206 and CD31 mRNA levels in melanoma patients (n = 322) (Figure S1G). These data support the notion that these non-cancerous cellular components are upregulated in human melanoma.

Overall, the IFs of human melanomas are enriched in amoeboid melanoma cells, which are associated with a specific TME, the *amoeboid-associated TME* (AATME). Importantly, the TME found in metastatic sites mirrors the TME found in the IFs of melanomas, that is, the AATME (Figure 1K).

### Myosin II Activity in Melanoma Cells Favors Secretion of Immunomodulatory Factors

**Figure 2:**
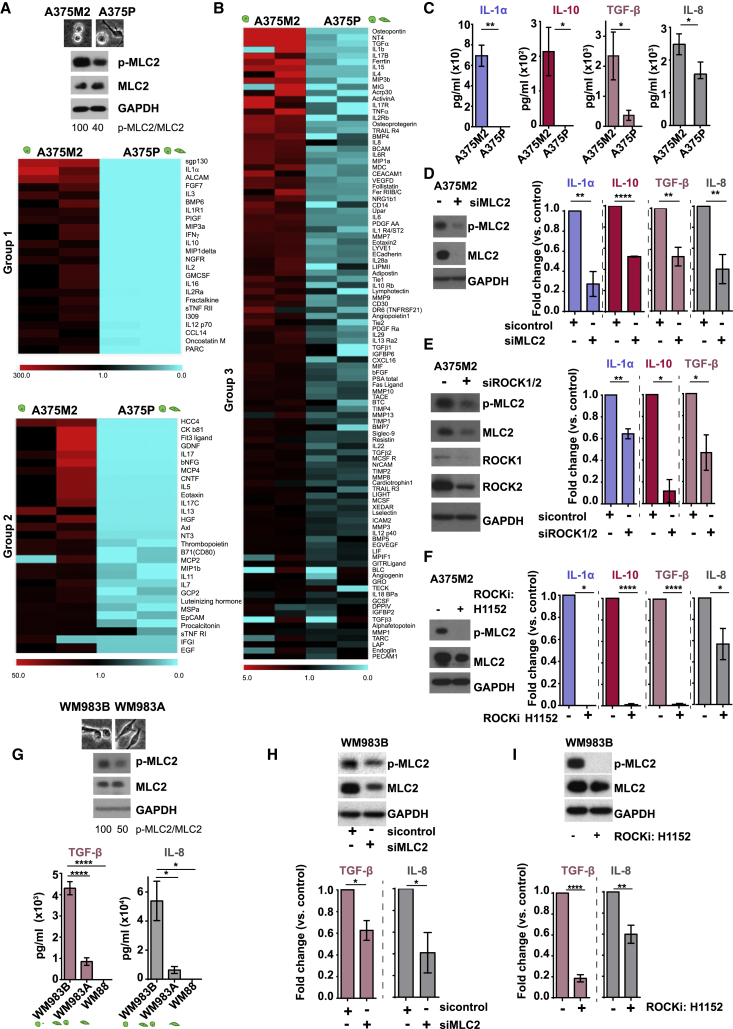
Myosin II Activity in Melanoma Cells Favors Secretion of Immunomodulatory Factors (A) (Top) Images and (bottom) immunoblotof p-MLC2 levels in A375M2 and A375P cells. (B) Heatmaps of secreted factors enriched in CM A375M2 with a >1.1 fold-increase compared to CM A375P, divided into 3 groups (0- to 300-, 0- to 50-, and 0- to 5-fold). Cyan and red indicate the lowest and highest expression levels, respectively. (C) Concentration of IL-1α, IL-10, TGF-β, and IL-8 in CM A375P or CM A375M2, by ELISA (n = 3). (D) After MLC2 knockdown in A375M2 cells, (left) representative immunoblot for p-MLC2 levels and (right) secreted levels of IL-1α, IL-10, TGF-β, and IL-8 in CM A375M2, by ELISA (n ≥ 3 for IL-1α, IL-8, and TGF-β, n = 2 for IL-10). (E) After ROCK1/2 knockdown in A375M2 cells, (left) representative immunoblots for ROCK1/2 and p-MLC2 levels and (right) secreted levels of IL-1α, IL-10, and TGF-β in CM A375M2 by ELISA (n ≥ 3 for IL-1α and TGF-β, n = 2 for IL-10). (F) After treatment with H1152 (5 μM) for 48 h in A375M2 cells, (left) representative immunoblot for p-MLC2 levels and (right) secreted levels of IL-1α, IL-10, TGF-β, and IL-8 in CM A375M2 by ELISA (n ≥ 3). (G) (Top) Images and immunoblot for p-MLC2 levels in WM983B and WM983A cells and (bottom) secreted levels of TGF-β and IL-8 in CM WM983B, CM WM983A, and CM WM88, by ELISA (n = 3 for all, n = 2 for IL-8 in CM WM88). (H) After MLC2 knockdown in WM983B cells, (top) representative immunoblot for p-MLC2 levels and (bottom) secreted levels of TGF-β and IL-8 in CM WM983B as tested by ELISA (n = 3). (I) After treatment with H1152 (5 μM) for 48 h in WM983B cells, (top) representative immunoblot for p-MLC2 levels and (bottom) secreted levels of TGF-β and IL-8 in CM WM983B (n ≥ 3). In (D)–(F), (H), and (I), data are presented as fold change versus the control. In (C)–(I), graphs show mean ± SEM. In (C)–(F), (H), and (I), t test is shown. In (G), one-way ANOVA with Tukey post hoc test is shown. ^∗^p < 0.05, ^∗∗^p < 0.01, ^∗∗∗∗^p < 0.0001. See also Figure S3 and Table S1.

**Figure S2:**
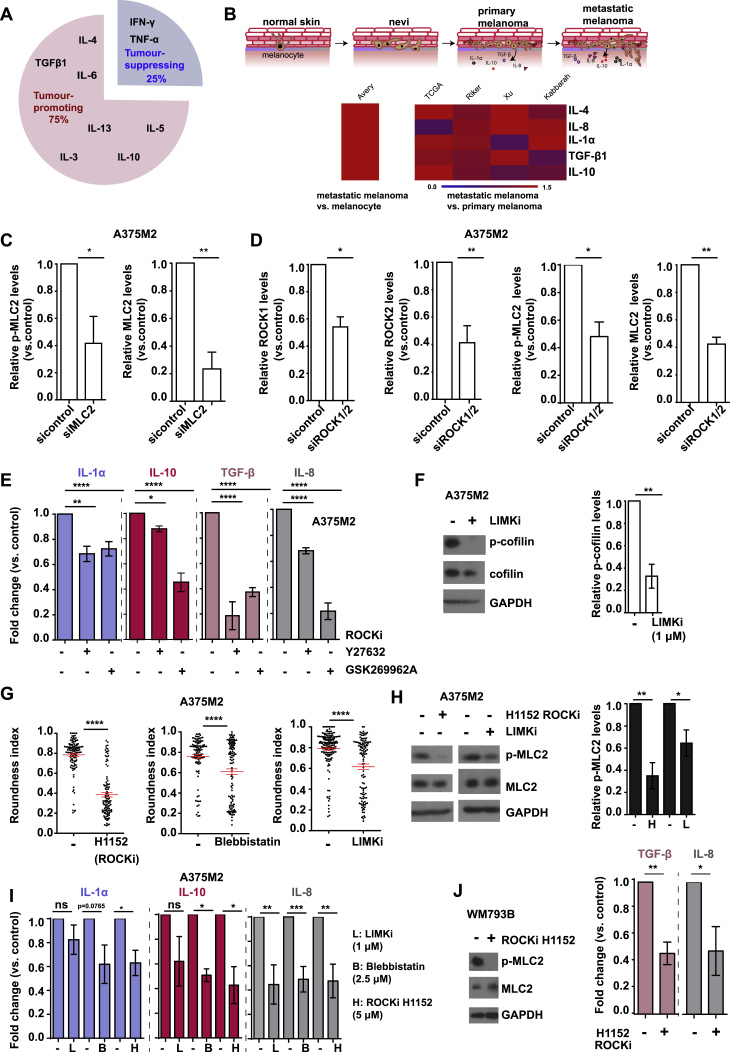
Myosin II Activity in Melanoma Cells Favors Secretion of Immunomodulatory Factors, Related to Figure 2 (A) Chart pie shows the factors influencing the balance toward tumor-promoting inflammation versus tumor-suppressive inflammation in CM A375M2. (B) (Top) Schematic illustrates secreted factors in melanoma progression and (bottom) heatmap shows fold change for mRNA levels of IL-4, IL-8, IL-1α, TGF-β and IL-10 in metastatic melanoma versus melanocyte and metastatic versus primary melanoma samples. Raw data were obtained from TCGA and GEO databases. (C) Relative p-MLC2 and MLC2 levels in A375M2 cells after MLC2 knockdown. (D) Relative ROCK1, ROCK2, p-MLC2 and MLC2 levels in A375M2 cells after ROCK1/2 knockdown. (E) Secreted levels of IL-1α, IL-10, TGF-β and IL-8 in CM A375M2 cells after treatment with Y27632 (10 μM) or GSK269962A (5 μM) for 48h (n ≥ 3). Data are presented as fold change versus the control. (F) (Left) Representative immunoblot for p-cofilin and (right) relative p-cofilin levels, in A375M2 cells after treatment with LIMKi 3 (1μM) for 48h (n = 3). (G) Roundness index of A375M2 cells seeded on top of collagen I, treated with H1152 (5μM), Blebbistatin (2.5μM) or LIMKi 3 for 48h (n = 3). (H) (Left) Representative immunoblots for p-MLC2 and (right) quantification of p-MLC2 levels, in A375M2 cells treated with H1152 or LIMKi 3 for 48h (n = 3). (I) Secreted levels of IL-1α, IL-10 and IL-8 in CM A375M2+H1152, CM A375M2+Blebbistatin or CM A375M2+LIMKi 3. Data are presented as fold change versus the control (n ≥ 3 for IL-10 and IL-8 and n ≥ 2 for IL-1α). (J) (Left) Representative immunoblot for p-MLC2 levels in WM793B cells and (right) secreted levels for TGF-β and IL-8 by WM793B cells, after treatment with H1152 (5 μM) for 48h (n ≥ 3).(C-J) Graphs and dot blots show mean ± SEM. (C, D, and F–J) t test. (E) One-way ANOVA with Tukey post hoc test. ns p > 0.05,^∗^p < 0.05,^∗∗^p < 0.01,^∗∗∗^p < 0.001,^∗∗∗∗^p < 0.0001.

Tumor cell-normal cell communication can be mediated by secreted factors (Melnikova and Bar-Eli, 2009). A375M2 are highly metastatic (Clark et al., 2000) rounded melanoma cells (∼90% rounded [Orgaz et al., 2014]) with higher Myosin II activity (Figure 2A). A375M2 cells are derived from poorly metastatic A375P (Clark et al., 2000) more elongated melanoma cells (50% rounded, 50% elongated [Orgaz et al., 2014]) with lower Myosin II activity compared to A375M2 cells (Figure 2A). Using a protein array consisting of 274 human chemokines, cytokines, growth factors, and matrix metalloproteinases, we found that 155 proteins were highly secreted by A375M2 cells compared to A375P cells (Figure 2B). These factors were sub-divided into 3 groups based on their fold change (Figure 2B). A375M2 cells were shown to secrete high levels of cytokines, such as IL-3, IL-4, IL-5, and IL-13. The amoeboid-melanoma secretome appears to be skewed toward a pro-inflammatory signature typically associated with tumor progression (Figure S2A). We confirmed by ELISA that A375M2 cells secreted high levels of pro-inflammatory IL-1α, IL-8, and immunosuppressive IL-10 and TGF-β (Figure 2C). To expand our observations to the clinical setting, GEO (n = 421) and TCGA (n = 354) databases were used to evaluate mRNA levels of some highly secreted factors by A375M2 cells. IL-1α, IL-10, TGF-β, IL-8, and IL-4 mRNA were all upregulated during melanoma progression with a significant increase in metastatic compared to primary human melanomas (Figure S2B) suggesting transcriptional regulation.

We confirmed that protein secretion was Myosin II dependent since A375M2 cells depleted from MLC2 secreted significantly less cytokines and chemokines (Figures 2D and S2C). ROCK is a key regulator of Myosin II activity (Amano et al., 1996), and, as such, A375M2 cells depleted from ROCK1/2 via RNAi had decreased protein secretion (Figures 2E and S2D). These results were confirmed using 3 ROCK inhibitors (ROCKi H1152, Y27632, GSK269962A) (Figures 2F and S2E). Moreover, inhibition of LIMK downstream of ROCK in A375M2 cells resulted in reduced: p-cofilin levels (Figure S2F), cell roundness (Figure S2G), p-MLC2 levels (Figure S2H), and IL-8 secretion (Figures S2I). These data indicate that perturbing actin and myosin dynamics has an impact on secretion.

Our observations were further expanded to the matched melanoma paired cell lines WM983B (metastatic) /WM983A (primary) derived from the same patient (Cantelli et al., 2015). WM983B cells were more rounded with higher p-MLC2 levels compared to WM983A cells (Figure 2G, upper panels) and were found to be more secretory (Figure 2G, lower panels). On the other hand, WM88 elongated melanoma cells (Cantelli et al., 2015) did not secrete any of the cytokines measured (Figure 2G). To further asses the role of ROCK-Myosin II, WM983B cells were depleted from MLC2 (Figure 2H, upper panel) or treated with a ROCKi (Figure 2I, upper panel) and were found to secrete significantly less cytokines (Figures 2H and 2I, lower panels). Similar results were observed when ROCK was inhibited in an additional melanoma cell line WM793B (Figure S2J).

Overall, our data show that the secretion of immunomodulatory factors, which are important during melanoma progression, is regulated by ROCK-Myosin II activity in melanoma cells.

### Amoeboid Melanoma Cells Induce Tumor-Promoting Macrophages

**Figure 3:**
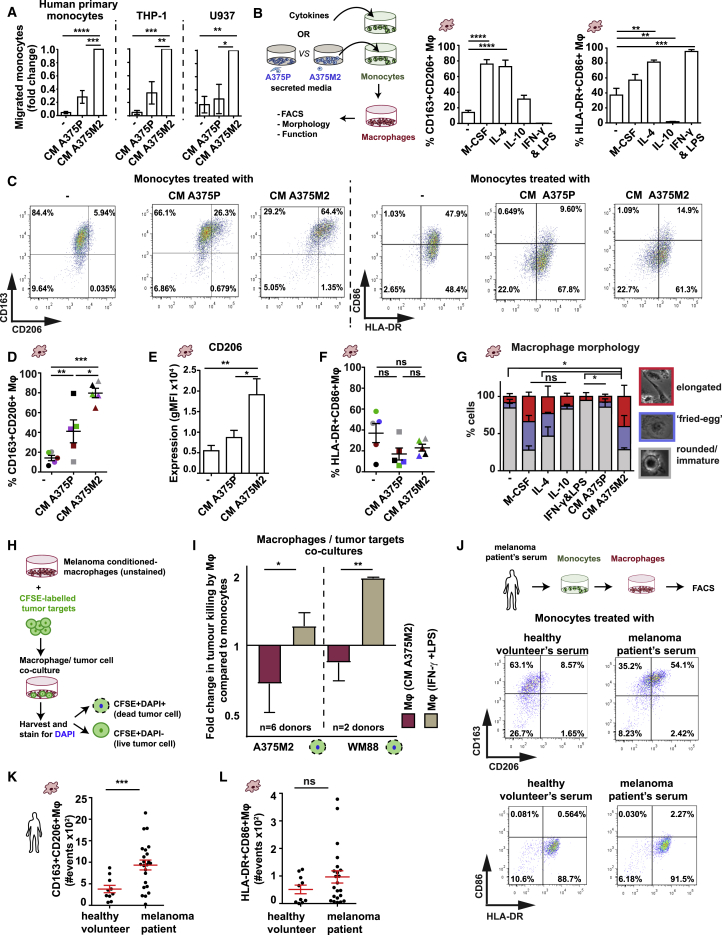
Amoeboid Melanoma Cells Induce Tumor-Promoting Macrophages (A) Percentage of migrated human PBMC-derived monocytes, THP-1 and U937 toward media (–), CM A375P, or CM A375M2 (n = 3). (B) (Left) Schematic: *in-vitro*-polarized macrophages or melanoma-conditioned macrophages and (right) %CD163^+^CD206^+^ or %HLA-DR^+^CD86^+^ macrophages after treatment with M-CSF, IL-4, IL-10, or IFN-γ&LPS or media only (–) (n = 5;5 different healthy donors). (C) Fluorescence-activated cell sorting (FACS) dot plots from one donor showing (left) %CD163^+^CD206^+^ and (right) %HLA-DR^+^CD86^+^ macrophages after treatment with CM A375P, CM A375M2 or culture media only (–). (D–F) %CD163^+^CD206^+^ macrophages (D), mean fluorescence intensity (MFI) for CD206 (E), and %HLA-DR^+^CD86^+^ macrophages (F), after treatment with CM A375P, CM A375M2, or culture media only (–) (n = 5;5 different healthy donors). (G) Quantification of macrophage morphology (see also Figure S3D) (n = 3;3 different healthy donors). (H) Schematic shows macrophage cytotoxicity assay. (I) Fold change of dead tumor cells (A375M2 or WM88) in co-cultures with PBMC-derived monocytes treated with CM A375M2 or IFN-γ&LPS. Data are presented as fold change versus the control untreated monocytes. Log2 scale is presented in y axis (n = 6;6 different healthy donors for A375M2 co-cultures and n = 2;2 different healthy donors for WM88 co-cultures). (J) (Top) Schematic shows TAMs induction *in vitro* with serum from melanoma patients and (bottom) representative FACS dot plots from one donor showing %CD163^+^CD206^+^ and %HLA-DR^+^CD86^+^ macrophages after treatment with healthy volunteer’s serum or melanoma patient’s serum. (K and L) Number of (K) CD163^+^CD206^+^ and (L) HLA-DR^+^CD86^+^ macrophages after treatment with healthy volunteer’s serum or melanoma patient’s serum (n = 3;3 different healthy donors. Sera from n = 10 healthy volunteers, n = 23 melanoma patients, each dot represents a different treatment). In (A), (B), (D)–(G), (I), (K), and (L), graphs and dot blots show mean ± SEM. In (A), (B), and (D)–(G), one-way ANOVA with Tukey or Bonferroni (for G) post hoc tests are shown. In (I), t test is shown. In (K) and (L), t test with Welch’s correction is shown. Nonsignificant p > 0.05, ^∗^p < 0.05, ^∗∗^p < 0.01, ^∗∗∗^p < 0.001, ^∗∗∗∗^p < 0.0001. See also Figure S3 and Tables S1 and S2.

Enrichment in amoeboid melanoma cells and TAMs in the IF of human melanomas (Figure 1) suggests a potential communication between cancer cells and macrophages. On the other hand, the secretome of amoeboid melanoma cells is rich in over 20 chemotactic factors (Table S1) known to trigger chemotaxis in monocytes, which are the precursors of macrophages. Indeed, migration of human peripheral blood mononuclear cell (PBMC)-derived monocytes and monocytic cell lines (THP-1 and U-937) was increased toward secreted factors (conditioned media [CM]) derived from amoeboid A375M2 versus more elongated A375P cells (Figure 3A). The timescales of this assay (2–4 h) (Ancuta et al., 2003) suggest that increased chemotaxis was responsible for increased monocytic migration.

**Figure S3:**
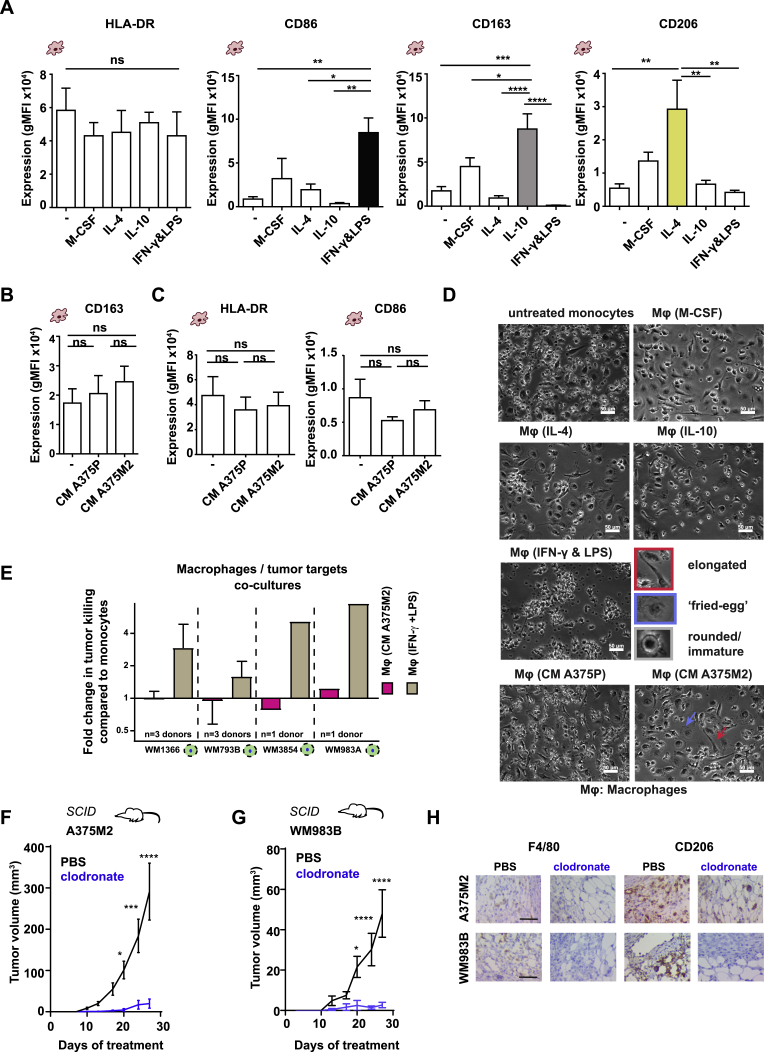
Amoeboid Melanoma Cells with High Myosin II Activity Induce Tumor-Promoting Macrophages, Related to Figure 3 (A) Expression levels (geometric mean of fluorescent intensity, gMFI) of HLA-DR, CD86, CD163 and CD206 in macrophages after treatment with M-CSF (50ng/ml), IL-4 (20ng/ml), IL-10 (20ng/ml), IFN-γ & LPS (20ng/ml and 100ng/ml) or culture media only (-) (n = 5; 5 different healthy donors). (B and C) Expression levels (gMFI) of (B) CD163, (C) HLA-DR and CD86, in macrophages treated with CM A375P or CM A375M2 or culture media only (-) (n = 5; 5 different healthy donors). (D) Representative bright-field images of macrophages treated with M-CSF, IL-4, IL-10, IFN-γ & LPS or culture media only (-). Blue and red arrows show ‘fried-egg’ and elongated shapes, respectively. Scale bar, 50 μm. (E) Dead tumor targets (WM1366, WM793B, WM3854 and WM983A) upon co-culture with CM A375M2- or IFN-γ&LPS- stimulated macrophages. Data are presented as fold change versus the control untreated monocytes. Log2 scale is presented in y axis. (F and G) Tumor volume in (F) A375M2-xenografts and (G) WM983B-xenografts, upon depletion of macrophages via clodronate in SCID mice (n = 6 mice PBS group and n = 5 mice clodronate group). (H) Representative IHC images showing F4/80+ and CD206+ macrophage depletion upon clodronate administration in A375M2 and WM983B xenografts. Scale bar, 100 μm. (A–C and E–G) Graphs show mean ± SEM. (A–C) One-way ANOVA with Tukey post hoc test. (F and G) Two-way ANOVA with Bonferroni’s multiple comparison test. ns p > 0.05,^∗^p < 0.05,^∗∗^p < 0.01,^∗∗∗^p < 0.001,^∗∗∗∗^p < 0.0001.

We next addressed whether monocytes could be differentiated into macrophages in response to CM A375M2, as this media was rich in factors (macrophage colony stimulating factor [M-CSF], IL-4, IL-10, IL-13, TGF-β) that affect monocyte-macrophage commitment. We developed a spectrum of *in-vitro*-polarized macrophages (Gordon, 2003, Mantovani et al., 2004) (Figure 3B). M-CSF, IL-4, or IL-10 treatment showed increased CD163^+^CD206^+^ expression in macrophages, while HLA-DR^+^CD86^+^ macrophages were induced after IFN-γ&LPS (Figure 3B). Specifically, IL-4 induced CD206, IL-10 induced CD163, IFN-γ&LPS induced CD86 expression, while HLA-DR expression was similar across treatments (Figure S3A). Importantly, CM A375M2 induced CD163^+^CD206^+^macrophages more efficiently than CM A375P (Figures 3C–3E and S3B). CD206 expression was increased in macrophages induced by CM A375M2 (Figure 3E) and comparable to that after IL-4 stimulation (Figure S3A). Neither CM A375M2- nor CM A375P-treated macrophages showed difference in expression levels of classic activation markers: HLA-DR and CD86 (Figures 3F and S3C). Macrophages induced by CM A375M2, IL-4, M-CSF, or IL-10 retained a mixed morphology (Figures 3G and S3D) indicative of differentiation (Eligini et al., 2013). Cell elongation, characteristic of AAMs (McWhorter et al., 2013), was profound in CM A375M2-treated macrophages, while minimal after IFN-γ&LPS treatment or in untreated monocytes (Figure 3G).

To characterize the functional role of macrophages-induced by amoeboid melanoma cells, a tumor cell-killing assay was performed (Figure 3H). IFN-γ&LPS-treated macrophages killed both amoeboid A375M2 and elongated WM88 cells (Figure 3I). In contrast, macrophages induced by CM A375M2 supported tumor cell viability (Figure 3I). Such observations were expanded to a wider panel of melanoma cells (WM1366, WM793B, WM3854, and WM983A) (Figure S3E). To further validate the tumor-promoting role of macrophages-induced by amoeboid melanoma cells, *in vivo* experiments were performed using A375M2 and WM983B xenografts in SCID mice. Macrophage depletion after clodronate liposome delivery (van Rooijen et al., 1996) resulted in impaired *in vivo* tumor growth in both A375M2 and WM983B tumors (Figures S3F and S3G). Depletion of F4/80^+^CD206^+^ cells was confirmed (Figure S3H). These data suggest a key role for macrophages in mediating melanoma tumor growth.

We next used serum derived from melanoma patients or healthy donors to treat PBMC-derived monocytes (Figure 3J). Melanoma-patient-derived sera induced higher levels of CD163^+^CD206^+^ macrophages compared to healthy-donor-derived sera, while HLA-DR and CD86 expression remained unchanged (Figures 3J–3L). Thus, melanoma-patient-derived secreted factors induce macrophage polarization comparable to amoeboid melanoma cells.

Overall, our data show that amoeboid melanoma cells with high Myosin II activity can recruit monocytes, differentiate them into macrophages, and functionally educate them to support tumor growth.

### AATME Composition Is a Conserved Feature in Melanoma *In Vivo*

**Figure 4:**
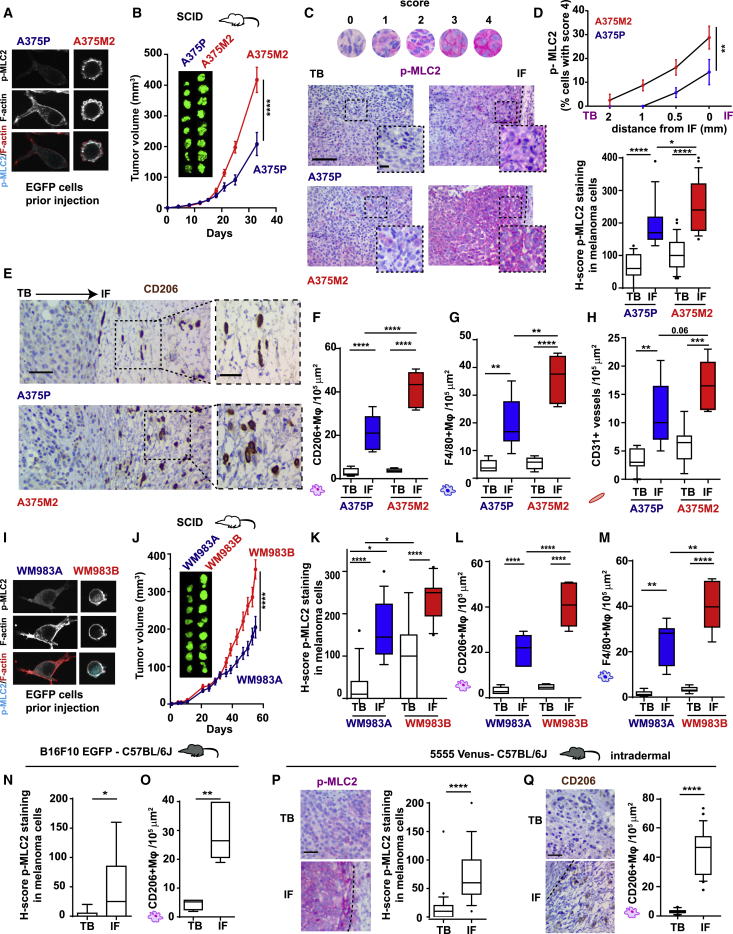
AATME Composition Is a Conserved Feature in Melanoma *In Vivo* (A) Confocal images for p-MLC2 (cyan) and F-actin (red) in EGFP-A375P and EGFP-A375M2 cells. (B) Tumor volume of xenografts post-injection of EGFP-A375P and EGFP-A375M2 cells (n = 8 mice/group). (C) Immunohistochemistry (IHC) images of p-MLC2 levels (scale bar, 50 μm; insert, 10 μm). (D) (Top) IHC quantification for p-MLC2 levels showing percentage of melanoma cells with the highest score (4) at different distances from IF (0–2 mm) and (bottom) H-score for p-MLC2 staining for A375P and A375M2 tumors (n = 8 mice/group). (E) Representative IHC images of CD206^+^ macrophages (scale bar, 30 μm and insert: 10 μm). (F–H) Quantification of (F) CD206^+^, (G) F4/80^+^, and (H) CD31^+^ cells in A375P and A375M2 tumors (n = 8 mice/group). (C)–(H) correspond to TB and IF of A375P and A375M2 xenografts, as tested by IHC (n = 8 mice/group). (I) Confocal images for p-MLC2 (cyan) and F-actin (red) in EGFP-WM983A and EGFP-WM983B cells. (J) Tumor volume of xenografts over time (35 days) post-injection of EGFP-WM983A and EGFP-WM983B cells (n = 8 mice/group). (K–M) H-score for p-MLC2 staining (K), quantification of CD206^+^ (L), and F4/80^+^ macrophages (M) in TB and IF of WM983A and WM983B xenografts, as tested by IHC (n = 8 mice/group). (N and O) H-score for p-MLC2 (N) and quantification (O) of CD206^+^ macrophages in TB and IF of B16F10 tumors, as tested by IHC (n = 8 mice). (P and Q) (Left) IHC images and (right) H-score for p-MLC2 staining (P), (left) IHC images and (right) quantification of CD206^+^macrophages (Q) in TB and IF of 5555 tumors (n = 5 tumors). Scale bar, 30 μm. In (B), (D, top), and (J), graphs show mean ± SEM. In (D, bottom), (F)–(H), and (K)–(Q), boxplots show 10–90 percentile. In (B), (D, top), and (J), two-way ANOVA with Bonferroni post hoc test is shown. In (D, bottom), (F)–(H), and (K)–(M), one-way ANOVA with Tukey post hoc test is shown. In (N)–(Q), t test is shown. Nonsignificant p > 0.05, ^∗^p < 0.05^∗∗^, p < 0.01, ^∗∗∗^p < 0.001, ^∗∗∗∗^p < 0.0001. See also Figure S4.

**Figure S4:**
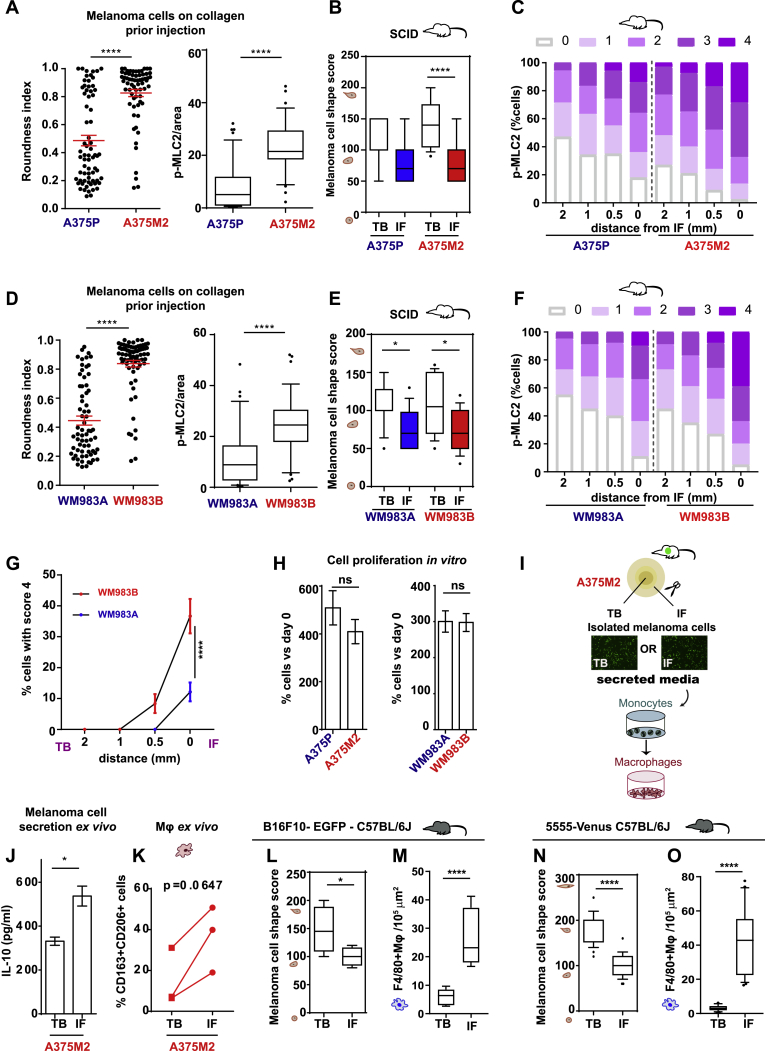
AATME Composition Is a Conserved Feature in Melanoma *In Vivo*, Related to Figure 4 (A) (Left) Roundness index and (right) p-MLC2 levels/area, in EGFP-A375P and EGFP-A375M2 cells seeded on top of collagen I. Quantification corresponds to the area occupied by p-MLC2 staining normalized by the total area of the cell. (B) Melanoma cell shape score in TB and IF of A375P and A375M2 tumors (n = 8 mice/group). (C) Percentage of melanoma cells with score 0-4 for p-MLC2 at different distance from IF (0-2 mm) for A375P and A375M2 tumors, as tested by IHC (n = 8 mice/group). (D) (Left) Roundness index and (right) p-MLC2 levels/area, in EGFP-WM983A and EGFP-WM983B cells seeded on top of collagen I. (E) Melanoma cell shape score in TB and IF of WM983A and WM983B tumors (n = 8 mice/group). (F) Percentage of melanoma cells with score 0-4 for p-MLC2 at different distance from IF (0-2 mm) for WM983A and WM983B tumors (n = 8 mice/group). (G) Percentage of melanoma cells with score 4 for p-MLC2 at different distance from IF (0-2 mm) for WM983A and WM983B tumors, as tested by IHC (n = 8 mice/group). (H) Cell proliferation rates of (left) A375P versus A375M2 and (right) WM983A versus WM983B cells. Data are presented as fold change versus day 0. (I) Schematic shows the isolation of melanoma cells from TB or IF of A375M2 tumors. (J) Concentration of secreted IL-10, as measured by ELISA, in melanoma cells isolated from TB or IF of A375M2 tumors (n = 3). (K) Percentage of CD163+CD206+ macrophages after treatment with CM from melanoma cells isolated from TB or IF of A375M2 tumors (n = 2; 2 different healthy donors; 2 matched TB/IF samples; 1 pair used twice with 2 different donors). (L and M) (L) Melanoma cell shape and (M) F4/80+ macrophages, in TB and IF of B16F10 tumors (n = 8 mice). (N) Melanoma cell shape score and (O) F4/80+ macrophages, in TB and IF of tumors generated 8 days post-intradermal injection of Venus- 5555 cells (n = 5 tumors). (A left, D left, G, and H) Graphs and dot blots show mean ± SEM. (A right, B, D right, E, and L–O) Boxplots show 10-90 percentile. (A, D, H, J, L–O) t test. (K) paired *t*- test. (B and E) One-way ANOVA with Tukey post hoc test. (G) Two-way ANOVA with Bonferroni post hoc test. ns p > 0.05,^∗^p < 0.05,^∗∗^p < 0.01,^∗∗∗^p < 0.001,^∗∗∗∗^p < 0.0001

We next assessed whether intrinsically high Myosin II activity in melanoma cells has an impact on macrophage recruitment *in vivo*. A375M2-EGFP cells (amoeboid and higher Myosin II activity) or A375P-EGFP cells (more elongated and lower Myosin II activity) (Figures 4A and S4A) were injected subcutaneously into SCID mice. A375M2 tumors grew faster compared to A375P ones (Figure 4B). There was an increase in melanoma cell roundness at the IFs of all tumors (Figure S4B). We assigned scores from 0 (low intensity) to 4 (very high intensity) of phospho-MLC2 (Figures 4C, 4D, and S4C). Overall, Myosin II levels were higher in A375M2 tumors compared to A375P tumors in all areas (Figures 4C, 4D, upper panel, and S4C). Importantly, Myosin II activity was significantly increased in all the IFs (Figures 4C and 4D), while we detected the highest levels of Myosin II activity at the IF of A375M2 tumors (Figures 4C, 4D, and S4C) accompanied by the highest infiltration of F4/80^+^CD206^+^ TAMs in conjunction with vessel density (Figures 4E–4H). Similarly, WM983B-EGFP cells (amoeboid, higher Myosin II) and WM983A-EGFP cells (more elongated, lower Myosin II) (Figures 4I and S4D) were injected into SCID mice. WM983B tumors grew faster compared to WM983A ones (Figures 4J). Melanoma cell rounding was observed at the IF (Figure S4E), while WM983B tumors had the highest levels of Myosin II (Figures 4K, S4F, and S4G). Similarly, WM983B tumors had the highest F4/80^+^CD206^+^ TAM infiltration at the IFs, compared to WM983A tumors (Figures 4L and 4M). Notably, A375P versus A375M2 and WM983A versus WM983B showed similar proliferation rates *in vitro* (Figure S4H). Since SCID mice are deficient in B and T cells, amoeboid melanoma cells could induce macrophage polarization *in vivo*. Altogether, these data suggest that invasive amoeboid melanoma cells with high Myosin II activity educate macrophages to jointly support tumor growth.

Next, we investigated whether amoeboid invasive melanoma cells could retain their secretory memory. Melanoma cells were isolated from the IFs and TBs of A375M2 tumors and cultured *ex vivo (*Figure S4I). Melanoma cells isolated from the IF of A375M2 tumors were more secretory (Figure S4J) and induced CD163^+^CD206^+^ macrophages more efficiently *ex vivo* when compared to their TB counterparts *(*Figure S4K).

Moreover, to ensure that AATME can be generated even in the presence of all immune components, highly metastatic and amoeboid B16F10 cells were injected into immunocompetent C57BL/6J mice. We observed rounded melanoma cells (Figure S4L) with high Myosin II levels (Figure 4N) and increased F4/80^+^CD206^+^ TAMs at the IFs (Figures 4O and S4M). To closely recapitulate the physiological development of melanoma TME, we used an orthotopic model in which amoeboid 5555 cells—derived from a BRAF^V600E^ mouse model (Dhomen et al., 2009)—were injected intradermally into C57BL/6J mice. As in previous models, IFs of 5555 tumors were rich in rounded cancer cells (Figure S4N) with increased Myosin II activity (Figure 4P) and F4/80^+^CD206^+^ TAM infiltration (Figures 4Q and S4O). Overall, these results suggest that AATME is a conserved feature in several *in vivo* melanoma tumor models.

### Blocking Myosin II Activity in Melanoma Cells Reprograms Macrophages

**Figure 5:**
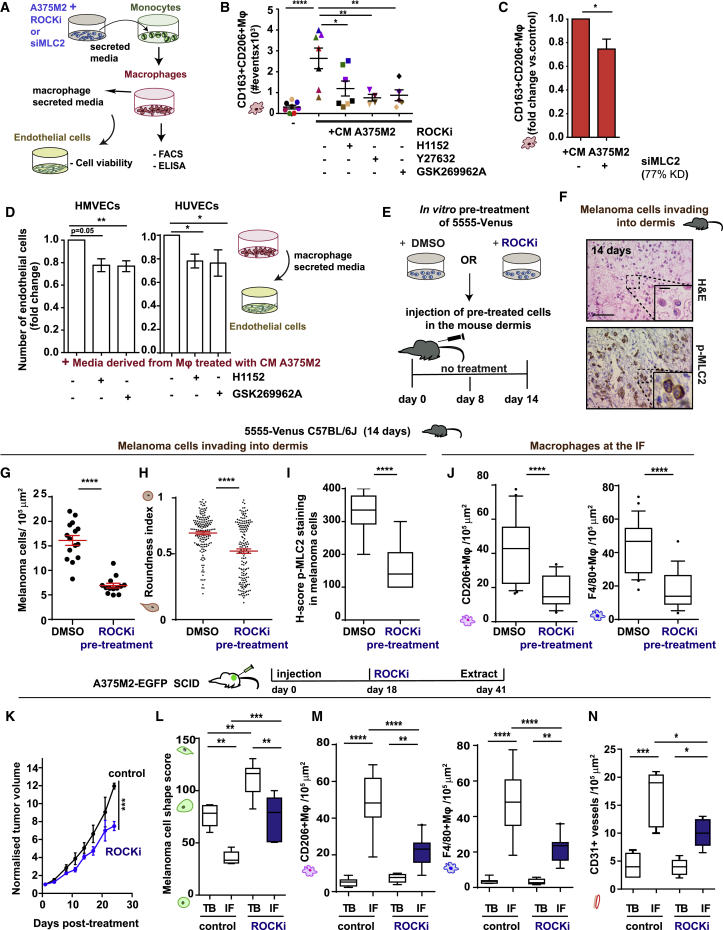
Blocking Myosin II Activity in Melanoma Cells Reprograms Macrophages (A) Schematic: *in vitro* treatment of PBMC-derived monocytes with CM from ROCK inhibited or MLC2 depleted A375M2 cells and subsequent assays. (B) CD163^+^CD206^+^ macrophages after treatment of PBMC-derived monocytes with CM A375M2, CM A375M2+ROCKi, or media only (–) (n = 8;8 different healthy donors; each dot is a different donor). (C) PBMC-derived monocytes treated with CM A375M2 depleted from MLC2 and quantification of CD163^+^CD206^+^ macrophages. Data are presented as fold changes versus control (n = 4). (D) Quantification of fold change of absorbance (O.D.): viability of endothelial cells (HMVECs and HUVECs) after treatment with macrophage-derived supernatants (50%). Media were derived from monocytes ± CM A375M2+ROCKi (n ≥ 3 for HMVECs and n = 6 for HUVECs). Endothelial cells were treated with macrophage-derived supernatants for 72 h. (E) Schematic: *in vivo* experiment with GSK269962A ROCKi pre-treated 5555 cells. (F) Representative H&E image (top) and IHC image for p-MLC2 showing amoeboid melanoma cells with high p-MLC2 invading the dermis in the IF of tumors 14 days post-intradermal injection of DMSO (vehicle) pre-treated 5555 cells. Scale bar, 50 μm; insert, 10 μm. (G–I) Number of invading melanoma cells (G), roundness index (H), and H-score (I) for p-MLC2 for (G). (J) (Left) CD206^+^ and (right) F4/80^+^ macrophages in the IF. (G–J) Tumors from DMSO (vehicle) pre-treated or ROCKi-pretreated Venus-5555 cells 14 days post-intradermal injection (n = 14 control and n = 12 ROCKi). (K) Tumor volume of A375M2 xenografts treated with PBS or Y27632 ROCKi. (L–N) Melanoma cell-shape score (L) and quantification (M) of (left) CD206^+^ and (right) F4/80^+^ and (N) CD31^+^ cells, in A375M2 xenografts treated with PBS or Y27632 ROCKi (n ≥ 4). In (B)–(D), (G), (H), and (K), graphs and dot blots show mean ± SEM. In (I), (J), and (L)–(N), boxplots show 10–90 percentile. In (B) and (L)–(N), one-way ANOVA with Tukey post hoc test is shown. In (C) and (G)–(J), t test is shown. In (D), Kruskal-Wallis test followed by Dunn’s multiple comparisons test is shown. In (K), two-way ANOVA with Bonferroni post hoc test is shown. Nonsignificant p > 0.05, ^∗^p < 0.05, ^∗∗^p < 0.01, ^∗∗∗^p < 0.001, ^∗∗∗∗^p < 0.0001. See also Figure S5.

**Figure S5:**
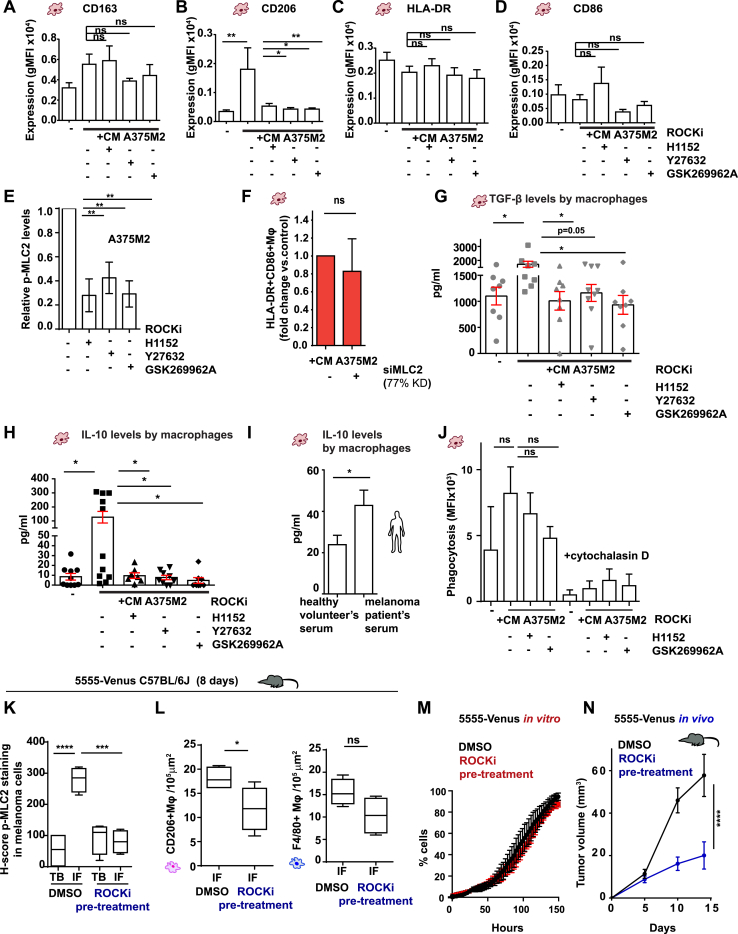
Remodeling of the AATME after Manipulation of Myosin II Activity *In Vivo*, Related to Figure 5 (A–D) Expression levels (gMFI) of (A) CD163, (B) CD206, (C) HLA-DR and (D) CD86, in macrophages after treatment with CM A375M2, CM A375M2+H1152, CM A375M2+Y27632, CM A375M2+GSK269962A or culture media only (-) (n ≥ 5 different heathy donors). (E) Relative p-MLC2 levels in A375M2 cells treated with H1152, Y27632 or GSK269962A ROCK inhibitors (n = 4). (F) HLA-DR+CD86+ macrophages after treatment with CM A375M2 depleted from MLC2. Data are presented as fold changes versus the control (n = 4). (G and H) (G) Macrophage secretion of TGF-β (pg/ml) and (H) IL-10 (pg/ml) after treatment with CM A375M2, CM A375M2+H1152, CM A375M2+Y27632, CM A375M2+GSK269962A or culture media only (-) (n = 8; 8 different healthy donors). (I) Concentration of IL-10 in macrophages induced by melanoma patient-derived sera or healthy volunteer-derived sera (n = 2; 2 different healthy donors for macrophages; n = 5 volunteers-derived sera and n = 9 melanoma patients-derived sera). (J) MFI for phagocytosed zymosan particles by macrophages after treatment with CM A375M2, CM A375M2+H1152 or CM A375M2+GSK269962A, with or without cytochalasin D (5 μM) (n = 2). (K) H-score for p-MLC2 expression of melanoma cells in TB and IF. (L) (Left) CD206+ macrophages and (right) F4/80+ macrophages in the IF. (K and L) Tumors 8 days post-intradermal injection of DMSO (vehicle)-pre-treated and ROCKi-pre-treated Venus- 5555 cells (n = 5 mice/group). (M) Percentage of viable DMSO (vehicle)-pre-treated and ROCKi-pre-treated Venus- 5555 cells *in vitro* as measured by IncuCyte (n = 3). Viability was measured after drug removal. (N) Tumor volume (mm^3^) in C57BL/6J mice after intradermal injection of DMSO (vehicle)-pre-treated and ROCKi-pre-treated Venus- 5555 cells (days: 0-14) (n = 13 tumors for DMSO group and n = 14 tumors for ROCKi group; n = 7 mice/group). (A–J, M, and N) Graphs show mean ± SEM. (K–L) Boxplots show 10-90 percentile. (A-E, J, and K) One-way ANOVA with Tukey post hoc test. (F–H and L) t test. (I) t test with Welch’s correction. (N) Two-way ANOVA with Bonferroni post hoc test. ns > 0.05,^∗^p < 0.05,^∗∗^p < 0.01,^∗∗∗^p < 0.001,^∗∗∗∗^p < 0.0001.

We next investigated whether ROCK-Myosin II axis in amoeboid cancer cells is responsible for polarizing macrophages. Monocytes were treated with CM from ROCK-inhibited A375M2 cells using 3 different ROCK inhibitors (Figure 5A). ROCK-inhibited A375M2 cells could not induce CD163^+^CD206^+^macrophages (specifically CD206 expression) compared to control (Figures 5B, S5A, and S5B). HLA-DR and CD86 expression did not change (Figures S5C and S5D). In all cases, ROCK-inhibited A375M2 cells had lowered p-MLC2 levels (Figure S5E). Similarly, A375M2 cells were less efficient in inducing CD163^+^CD206^+^macrophages after MLC2 RNAi transfection (Figure 5C), while HLA-DR^+^CD86^+^ macrophages did not change (Figure S5F).

TAMs can secrete immunosuppressive factors, such as TGF-β and IL-10 (Pollard, 2004). Macrophages induced by CM A375M2 secreted high levels of TGF-β and IL-10 (Figures S5G and S5H). Tumor necrosis factor (TNF)-α, a key feature of classically activated macrophages, was undetectable (data not shown). Furthermore, monocytes treated with melanoma-patient-derived sera differentiated into macrophages, which also secreted higher levels of IL-10 compared to monocytes treated with sera from healthy donors (Figure S5I), showing that this is a feature of melanoma-associated macrophages. Moreover, monocytes treated with CM from ROCK-inhibited A375M2 cells differentiated into macrophages that secreted significantly less TGF-β and IL-10 (Figures S5G and S5H). Of note, macrophages in all experimental conditions were competent for phagocytosis and sensitive to cytochalasin D (Figure S5J). These data confirm that ROCK-Myosin II activity in melanoma cells influences macrophage functional polarization.

We have shown in Figure 1 that AAMs are found in proximity of blood vessels and AAMs can promote endothelial cell survival (Chen et al., 2011). Indeed, macrophages induced by CM A375M2 sustained endothelial cell growth in HMVECs (human microvascular endothelial cells) and HUVECs (human umbilical vessel endothelial cells) more efficiently than macrophages treated with CM from ROCK-inhibited A375M2 cells (Figures 5D). Overall, our data show that decreasing Myosin II activity in cancer cells leads to phenotypic and functional macrophage reprograming.

To confirm the role of ROCK-Myosin II activity in controlling AATME *in vivo*, 5555 cells were pre-treated with ROCKi *ex vivo* and subsequently injected in the dermis of C57BL/6J mice without any further ROCKi treatment (Figure 5E). 8 days post-injection, we observed that control tumors generated AATME (Figures S5K and S5L). Importantly, ROCKi pre-treated 5555 cells were not able to increase Myosin II levels or recruit F4/80^+^CD206^+^ TAMs in the IFs of tumors as efficiently as controls (Figures S5K and S5L). 14 days post-injection, melanoma cells had invaded the dermis using amoeboid invasion (Figure 5F). At this time point, tumors in the ROCKi pre-treated group displayed pronounced loss of invading amoeboid melanoma cells (Figures 5G–5I) and a reduction in F4/80^+^CD206^+^ TAMs (Figure 5J). The two groups grew similarly *in vitro*, but ROCKi pre-treated group displayed a clear growth disadvantage *in vivo* (Figures S5M and S5N). These data suggest that Myosin II activity triggers AATME early on in tumorigenesis, while AATME is further supported during tumor development by amoeboid invasive melanoma behavior.

To extend our observations to the clinical setting, in which drugs are administered systemically in established tumors, mice harboring A375M2-EGFP tumors were treated with ROCKi (Figures 5K–5N). Similar to the pre-treatment setting, reduced tumor growth after systemic ROCKi treatment (Figure 5K) was associated with loss of melanoma cell roundness (Figure 5L), decreased F4/80^+^CD206^+^ TAM infiltration (Figure 5M), and decreased vasculature (Figure 5N) in the IFs.

Overall, we suggest that AATME composition *in vivo* is dependent on ROCK-Myosin II activity in melanoma cells located in the IF. Inhibition of ROCK-Myosin II in melanoma cells hinders the generation of tumor-supportive microenvironments.

### Myosin II Activity in Melanoma Cells Is Self-Perpetuated via Secreted IL-1α-Induced NF-κB Activation

**Figure 6:**
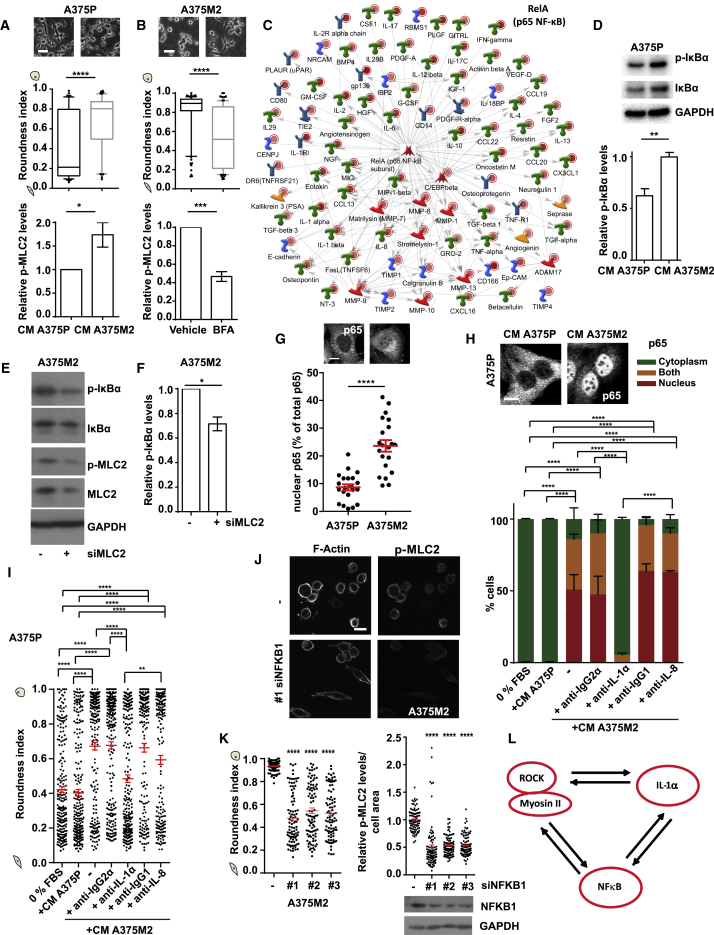
Myosin II Activity in Melanoma Cells Is Self-Perpetuated via Secreted IL-1α-Induced NF-κB Activation (A and B) (Top) Images, (center) roundness index, and (bottom) relative p-MLC2 levels of (A) A375P cells on top of collagen I upon treatment with CM A375P or CM A375M2 and (B) A375M2 cells upon treatment with BFA for 6 h. Data are presented as fold change versus the control (n = 3). (C) MetaCore enrichment network of factors upregulated in A375M2 cells is centered on NF-κB. (D) (Top) Immunoblot and (bottom) quantification of p-IKBα levels in A375P cells treated with CM A375P or CM A375M2. Data are presented as fold change versus CM A375M2 treatment (n = 3). (E and F) Immunoblot (E) and quantification (F) of p-IKBα levels in A375M2 cells after MLC2 knockdown. Data are presented as fold change versus control (n = 2). (G) (Top) Confocal images and (bottom) quantification of nuclear p65, as percentage versus the total p65, in A375P and A375M2 cells. Each dot represents a different cell. Scale bar, 10 μm. (H) (Top) Confocal images for p65 in A375P cells after treatment with CM A375P or CM A375M2. Scale bar, 10 μm. (bottom) Percentage of A375P cells with p65 in the cytoplasm (green), nucleus (red), or in both (orange) after indicated treatments. Blocking was for 1 h at 37°C (n = 3; 3 pictures per experiment; total 9 pictures per condition). (I) Roundness index of A375P cells on collagen I after same treatments as in (H) (n = 3). (J) Confocal images of p-MLC2 and F-actin in NFKB1 depleted A375M2 cells. Scale bar, 20 μm. (K) (Left) Roundness index and (right) relative p-MLC2 levels in A375M2 cells after NFKB1 knockdown, from confocal images (30 cells/condition, each dot represents a single cell). (K bottom right) Representative immunoblot showing NFKB1 knockdown in A375M2 cells. (L) Schematic: cross-talk between ROCK-Myosin II, secreted IL-1α, and NF-κB activation in amoeboid melanoma cells. In (A, center) and (B, center), boxplots show 10–90 percentile. In (A, bottom) and (B, bottom), (D), (F)–(I), and (K), graphs and dot blots show mean ± SEM. In (A), (B), (D), (F), and (G), t test is shown. In (H), (I), and (K), one-way ANOVA with Tukey post hoc test is shown. ^∗^p < 0.05, ^∗∗^p < 0.01, ^∗∗∗^p < 0.001, ^∗∗∗∗^p < 0.0001. See also Figure S6.

**Figure S6:**
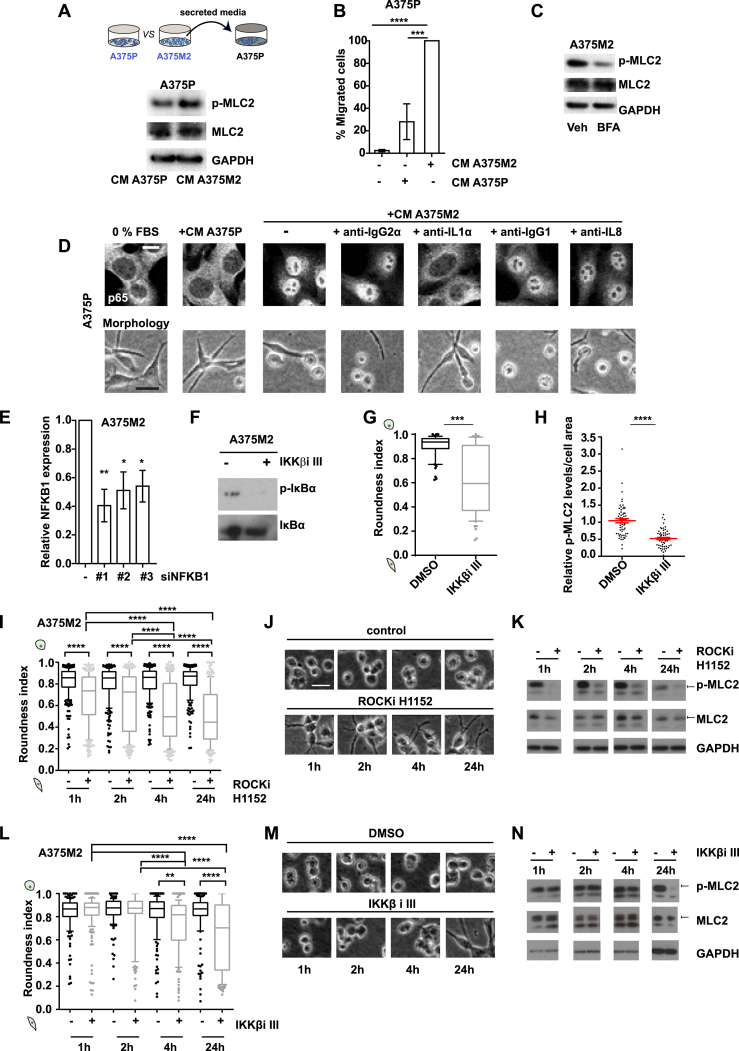
Myosin II Activity in Cancer Cells Self-Perpetuates via Secreted IL-1α-Induced NF-κB Activation, Related to Figure 6 (A) (Top) Schematic illustrates the treatment of A375P cells with CM A375M2 and (bottom) representative immunoblot for p-MLC2 in A375P cells after treatment with CM A375M2. (B) Percentage of migrated A375P cells after treatment with CM A375P, CM A375M2 or media only (-). Data are presented versus CM A375M2 treatment (n ≥ 3). (C) Representative immunoblot for p-MLC2 in A375M2 cells after treatment with BFA for 6h. (D) (Top) Representative confocal images and (bottom) bright-field images, showing p65 localization and cell morphology, respectively, in A375P cells treated with CM A375P, CM A375M2, IgG2α-blocked CM A375M2, IL-1α-blocked CM A375M2, IgG1-blocked CM A375M2 or IL-8-blocked CM A375M2. Blocking was for 1h at 37°C. Scale bar, 10μm for confocal images and 20μm for bright-field images. (E) Relative NFKB1 levels in A375M2 cells after NFKB1 knockdown. (F) Representative immunoblot for p-IκBα after treatment of A375M2 cells with IKKβ inhibitor. (G) Roundness index and (H) relative p-MLC2 levels, in A375M2 cells after treatment with IKKβ inhibitor. (I) Roundness index (n = 3), (J) representative bright-field images and (K) representative immunoblots for p-MLC2, in A375M2 cells seeded on bovine collagen I upon treatment with the ROCKi H1152 (5 μM) for 1h, 2h, 4h or 24h. (L) Roundness index (n = 3), (M) representative bright-field images and (N) representative immunoblots for p-MLC2, in A375M2 cells seeded on bovine collagen I upon treatment with the IKKβ inhibitor IKKβ III (0.5 μM) for 1h, 2h, 4h or 24h. (B, E, and H) Graphs and dot blots show mean ± SEM. (G, I, and L) Boxplots show 10-90 percentile. (B, E, I, and L) One-way ANOVA with Tukey post hoc test. (G and H) t test. ^∗^p < 0.05,^∗∗^p < 0.01,^∗∗∗^p < 0.001,^∗∗∗∗^p < 0.0001.

We have identified high levels of protein secretion as a characteristic of amoeboid melanoma cells. To assess whether protein secretion could affect Myosin II activity itself, A375P cells were treated with CM A375M2 and displayed increased: cell roundness (Figure 6A, upper panel), p-MLC2 levels (Figures 6A, lower panel, and S6A), and migration (Figure S6B). Thus, amoeboid melanoma cells exert paracrine effects toward other melanoma cells inducing amoeboid features via secretion. Next, A375M2 cells were treated with brefeldin A (BFA) to block trafficking of pre-stored soluble factors and receptors (Scales et al., 2000) and displayed loss of cell roundness (Figure 6B, upper panel) and p-MLC2 levels (Figures 6B, lower panel, and S6C). Thus, protein secretion sustains amoeboid features in an autocrine manner.

To understand which signaling pathways were linked to the amoeboid secretory phenotype, GeneGo MetaCore analysis was performed using the protein array data (Figure 2B). Amoeboid melanoma cells secrete factors that are part of a network centered on NF-κB (Figure 6C), while the second enriched network was centered on STAT3 (data not shown). NF-κB family members RELA (p65), RELB, and REL (c-Rel) contain transactivation domains. NFKB1 (p105) and NFKB2 (p100) encode longer proteins processed to the shorter DNA-binding forms p50 and p52. Phosphorylation of IκBα by the IKK complex enables dissociation from the NF-κB complex (p65/p50) with nuclear translocation of the latter (Perkins, 2012). To test whether protein secretion mediates NF-κB activity, A375P cells were treated with CM from A375M2 cells and displayed increased p-IκBα levels (Figure 6D). Conversely, depletion of MLC2 in A375M2 cells led to decreased p-IκBα levels (Figures 6E and 6F) suggesting that Myosin II regulates NF-κB activity. Moreover, amoeboid A375M2 melanoma cells have intrinsically high NF-κB activity (p65 nuclear translocation) compared to more elongated A375P cells (Figure 6G). These data show that amoeboid cells sustain NF-κB activity in an autocrine and paracrine manner.

We have shown that IL-1α and IL-8 secretion is regulated by ROCK-Myosin II (Figure 2), while NF-κB can be activated by either of these factors (Grivennikov and Karin, 2010). We hypothesized that ROCK-Myosin II activity could regulate NF-κB activation via either IL-1α or IL-8. CM from A375M2 cells induced p65 nuclear translocation in A375P cells (Figures 6H and S6D, upper panel). However, blocking IL-1α, but not IL-8, in CM A375M2 abolished these effects in A375P cells (Figures 6H and S6D, upper panel). To further understand whether secreted IL-1α itself had a role in controlling amoeboid features, A375P cells were treated with CM A375M2 in which IL-1α had been blocked. Blocking secreted IL-1α led to loss of cell roundness induced by CM A375M2, whereas blocking IL-8 had no effect (Figures 6I and S6D, lower panel). These data show that Myosin II activity in amoeboid melanoma cells regulates NF-κB activation via secreted IL-1α and conversely, IL-1α perpetuates the amoeboid phenotype.

We have shown that IL-1α supports both NF-κB activity and amoeboid phenotype. We hypothesized that NF-κB itself transcriptionally controls expression of secreted factors in CM A375M2 (Figure 6C) that induce an amoeboid phenotype (Figure 6A). A375M2 cells depleted from NFKB1 lost cell roundness and p-MLC2 cortical levels (Figures 6J, 6K, and S6E). Blockade of IKKβ activity led to inhibition of IκBα phosphorylation in A375M2 cells (Figure S6F) and an IKKβ inhibitor (IKKβi) yielded similar results as NFKB1 RNAi depletion (Figures S6G and S6H). Thus, IKKβ/NF-κB supports Myosin II activity in amoeboid melanoma cells generating a positive feedback loop. To further understand this signaling network, we studied the kinetics of this process. Cell roundness and p-MLC2 levels were decreased in a time-dependent manner using either ROCKi (Figures S6I–S6K) or IKKβi (Figures S6L–S6N). However, ROCK inhibition resulted in earlier changes compared to IKKβ inhibition, suggesting that NF-κB affects Myosin II activity indirectly at later time points via transcriptional mechanisms controlling cytokine expression, while ROCK regulates Myosin II activity directly.

In summary, ROCK-Myosin II activity and NF-κB establish a positive feedback loop initiated by ROCK regulation of IL-1α and perpetuated and amplified by IL-1α/IKKβ/NF-κB supporting amoeboid behavior in return (Figure 6L).

### NF-κB Cross-Talk with ROCK-Myosin II in Amoeboid Melanoma Cells Educates the Tumor Microenvironment

**Figure S7:**
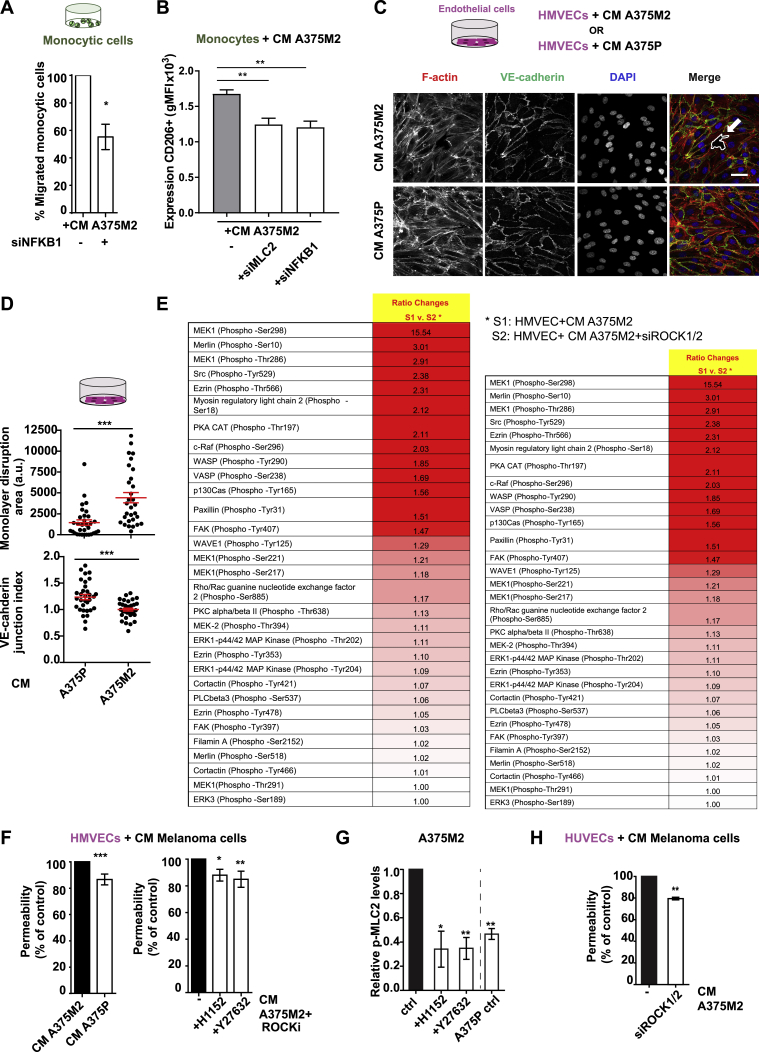
Metastatic Colonization via Amoeboid Melanoma Cell Secretion, Related to Figure 7 (A) Migrated THP-1 cells toward CM A375M2 or CM from NFKB1-depleted A375M2 cells (n = 4). (B) gMFI for CD206 in macrophages after treatment with CM from MLC2-depleted or NFKB1-depleted A375M2 cells (n = 4). (C) (Top) Schematic showing treatment of endothelial cells with CM A375P or CM A375M2. (Bottom) Representative confocal images of VE-cadherin (green), F-actin (red) and DAPI (blue) immunostaining in HMVECs after treatment with CM A375P or CM A375M2. Dashed white lines represent gaps. Scale bar, 40 μm. (D) Quantification of monolayer disruption area (top) and VE-cadherin junctional index (bottom) in HMVECs after treatment with CM A375P or CM A375M2 (n ≥ 3). (E) Tables show the most upregulated phospho-proteins found in the cytoskeleton phospho-antibody array. HMVECs treated with CM A375M2 (s1) were compared to HMVEC cells treated with CM from ROCK1/2-depleted A375M2 cells (s2). Values are represented as ratio changes s1/s2. Ratio = (Signal Intensity of Phospho Site-Specific Antibody) / (Signal Intensity of Site-Specific Antibody). Results are highlighted in different shades of red which shows the highest expression levels. Fold-change increase is considered significant when the values are > 2. (F) Percentage of permeability of a confluent monolayer of HMVECs (left) treated with CM A375P or CM A375M2 and (right) treated with CM A375M2+H1152 or CM A375M2+Y27632 (n ≥ 3). (G) Relative p-MLC2 levels in A375P cells, A375M2 ± H1152 or ± Y27632 cells (n ≥ 3). (H) Percentage of permeability of a confluent monolayer of HUVECs treated with CM A375M2 (-) or CM from ROCK1/2-depleted A375M2. Data are presented as fold-change versus the control (n = 3). (A, B, D, and F–H) Graphs and dot blots show mean ± SEM. (A, D, F left, and H) t test. (F right) Kruskal-Wallis and Dunn’s multiple comparison. (B and G) One-way ANOVA with Tukey post hoc test. ^∗^p < 0.05,^∗∗^p < 0.01,^∗∗∗^p < 0.001.

**Figure 7:**
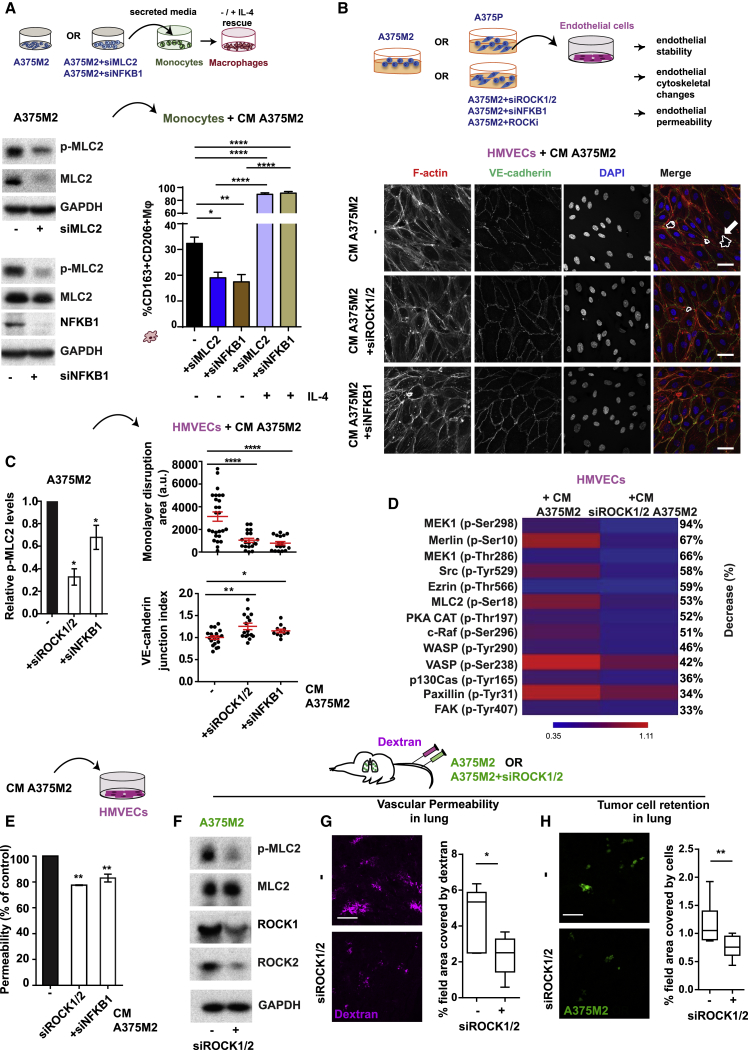
NF-κB Cross-Talk with ROCK-Myosin II in Amoeboid Melanoma Cells Educates the Tumor Microenvironment (A) (Top) Schematic: macrophage phenotypes after indicated treatments. (Bottom left) Immunoblots for p-MLC2 after MLC2 or NFKB1 knockdown in A375M2 cells. (Bottom right) Percentage of CD163^+^CD206^+^ macrophages upon treatment of PMBC-derived monocytes with CM from NFKB1-depleted or MLC2-depleted A375M2 cells, ±IL-4 (n = 4). (B) (Top) Schematic: treatment of endothelial cells with indicated conditions and downstream assays. (Bottom) Confocal images of VE-cadherin (green), F-actin (red), and DAPI (blue) immunostaining in HMVECs after indicated treatments. Dashed white lines represent gaps. Scale bar, 40 μm. (C) (Left) Relative p-MLC2 levels in indicated conditions (n ≥ 3). (Top right) Quantification of monolayer disruption area and (bottom right) VE-cadherin junctional index in HMVECs after indicated treatments (n ≥ 2). (D) Heatmap shows fold change in expression of proteins regulating endothelial permeability in HMVECs treated with CM A375M2 ± siROCK1/2. Blue and red show the highest and the lowest expression levels, respectively (6 replicates/antibody). (E) Permeability (versus the control) in HMVECs treated with indicated conditions (n ≥ 3). (F) Immunoblots for p-MLC2, ROCK1, and ROCK2 of A375M2 ± siROCK1/2. (G) (Left) Confocal images of mouse lungs after tail vein injection of 5-chloromethylfluorescein diacetate (CMFDA)-Green labeled A375M2 ± siROCK1/2 and dextran (purple) and (right) percentage of field area covered by dextran (20 fields/mouse/condition). (H) (Left) Confocal images of mouse lungs from (G) and (right) percentage of field area covered by cells (20 fields/mouse/condition). Scale bar in (G) and (H), 100 μm. In (G) and (H), n = 5 mice/condition for each experiment, n = 2 independent experiments. In (A), (C), and (E), graphs and dot blots show mean ± SEM. In (G) and (H), boxplots show min to max values. In (A), (C), and (E), one-way ANOVA and Tukey post hoc test are shown. In (G) and (H), t test is shown. ^∗^p < 0.05, ^∗∗^p < 0.01, ^∗∗∗∗^p < 0.0001. See also Figure S7.

We have shown that melanoma cells acquire amoeboid features that can be perpetuated by secretion via a cross-talk with NF-κB. Over 20 chemokines (Table S1) and several cytokines secreted by amoeboid cells were connected to NF-κB (Figure 6C) pointing at this transcription factor as a master regulator of the effects exerted by amoeboid cells on TME. In fact, NFKB1 depletion in A375M2 cells resulted in CM with decreased chemotactic potential toward monocytic cells (Figure S7A). Moreover, depletion of NFKB1 in amoeboid melanoma cells reduced Myosin II activity (Figure 7A, left panels), secretory potential and macrophage polarization (Figure 7A, right panel). These effects were comparable to MLC2 depletion via RNAi (Figure 7A). These data show that ROCK-Myosin II regulates macrophage polarization mainly via cytokines connected to NF-κB network (Figure 6C). Importantly, MLC2 and NFKB1 depletion in melanoma cells resulted in decreased CD206 in macrophages (Figure S7B). Among the cytokines in our panel, IL-4 induced the highest levels of CD206 expression (Figure S3A). Importantly, we could rescue the defects in macrophage polarization after NFKB1 or MLC2 depletion upon addition of IL-4 (Figure 7A, right panel). Therefore, the cross-talk between ROCK-Myosin II and NF-κB helps melanoma cells at the IF polarize macrophages via secreted factors.

Once amoeboid cells acquire high levels of Myosin II at the IF, they invade the dermis (Figures 5F and 5G). Such cancer cells could successfully colonize distant metastatic sites, where they need to extravasate (Reymond et al., 2013). At this stage, endothelial cell-cancer cell communication is crucial (Joyce and Pollard, 2009). Since the amoeboid secretome is rich in factors controlling vascular permeability (Figure 2B), we hypothesized that amoeboid cells could regulate the endothelium via secretion. In fact, abnormal endothelium and vessel leakiness are characterized by inter-endothelial gaps (Garcia et al., 1995, Hashizume et al., 2000). Endothelial cell monolayers recapitulating established blood vessels were treated with CM A375M2 or CM A375P (Figure S7C, upper panel), and inter-endothelial gap formation was favored when HMVECs were treated with CM A375M2 compared to CM A375P (Figures S7C and S7D). This was accompanied by decreased vascular endothelial (VE)-cadherin junctional index (Figures S7C and S7D, lower panel). CM A375M2-induced effects were lost if HMVECs were treated with CM derived from ROCK1/2-depleted or NFKB1-depleted A375M2 cells (Figures 7B and 7C, right panel). In these conditions, A375M2 had lowered Myosin II levels (Figure 7C, left panel).

Cytoskeletal changes in endothelial cells affect endothelial integrity (Giannotta et al., 2013). A phospho-antibody array (141 cytoskeletal regulators) was used to measure changes in endothelial cells (Figures 7D and S7E). HMVECs treated with CM A375M2 ROCK1/2-depleted cells showed decreased levels of phospho-proteins (Figures 7D and S7E) regulating vascular permeability (MEK, Src, PKA, and MLC2) (Kumar et al., 2009) were measured (Figures 7D and S7E). Furthermore, we found reduced endothelial permeability in HMVECs treated with CM from A375P cells compared to A375M2 cells (Figure S7F, left panel). Similar effects were observed in HMVECs when treated with CM from A375M2- ROCK1/2 or NFKB1-depleted (Figure 7E), or CM from A375M2 cells treated with ROCKi (Figure S7F, right panel). In all cases, Myosin II levels in melanoma cells were decreased (Figures 7C, 7F, and S7G). Similar effects were observed in HUVECs (Figure S7H). Therefore, cross-talk between ROCK and NF-κB in melanoma cells facilitates endothelial cell cytoskeletal remodeling.

Lung is one of the main sites where melanoma metastasizes (Obenauf and Massague, 2015), and lung retention assays have been used to measure metastatic colonization abilities (Medjkane et al., 2009, Orgaz et al., 2014). Importantly, melanoma cells with decreased ROCK-Myosin II (Figure 7F) could not alter the permeability of lung capillaries (Figure 7G) and were less efficient in colonizing the lung compared to controls (Figure 7H).

Thus, amoeboid melanoma cells with high Myosin II activity and high secretory potential have an advantage once they reach the metastatic site; they remodel the cytoskeleton of endothelial cells and increase vascular permeability to colonize the lung.

## Discussion

ROCK-Myosin II contributes to amoeboid tumor invasion and metastasis in a cell-autonomous manner (Sahai and Marshall, 2003, Sanz-Moreno et al., 2008, Sanz-Moreno et al., 2011). Here, we show that amoeboid melanoma cells educate both the myeloid and the endothelial compartment via secretion, regionally, in the IFs of tumors and later at distant metastatic sites. Thus, we define a new regulatory role for Myosin II dynamics in cancer cells beyond intrinsic control of cell motility.

AAMs compose the bulk of TAMs and are considered to be tumor promoting (Mantovani et al., 2002). Amoeboid cancer cells induce macrophages that are CD206^high^CD163^high^HLA-DR^+^CD86^low^ and produce IL-10 and TGF-β. Such macrophages support both melanoma cell growth and endothelial cell growth. The ratio of “amoeboid melanoma-induced macrophages” over “tumor-killing macrophages” will have a positive impact on tumorigenesis.

We show that amoeboid cancer cells retain their secretory potential at later metastatic stages, since they have an advantage during metastatic colonization via disrupting endothelial junctions and increasing endothelial cell permeability. Therefore, amoeboid cancer cells hijack these efficient mechanisms—typical of immune cells—to modify the vasculature (Artemenko et al., 2014).

The secretome of amoeboid melanoma cells is complex and rich in immunomodulatory cytokines, chemokines, and growth factors. We show that amoeboid behavior is sustained via a positive feedback loop between ROCK-Myosin-II-driven secretion and IL-1α/NF-κB signaling, generating a strong circuit of signal amplification. Depletion or inhibition of ROCK-Myosin II activity reduces (1) amoeboid behavior, (2) secreted IL-1α, and (3) NF-κB activation in melanoma cells. Conversely, targeting NF-κB in melanoma cells abolishes amoeboid behavior and their secretory profile resulting in defective macrophage polarization and vascular permeability. While targeting NF-κB in the clinic is challenging (Croghan et al., 2010, Markovic et al., 2005), ROCK inhibitors are used to treat cerebral vasospasm and intraocular pressure (Feng et al., 2016, Olson, 2008). ROCK inhibitors decrease invasion in pancreatic cancer (Rath et al., 2017) and improve efficacy of chemotherapy (Vennin and Chin, 2017). Moreover, ROCKi AT13148 (Sadok et al., 2015) is in clinical trials for solid tumors (ClinicalTrials.gov identifier: NCT01585701). In light of our findings, ROCK-Myosin II inhibition in melanoma cells could also be used to reprogram the innate immune microenvironment.

On the other hand, blocking secreted IL-1α in melanoma cells is sufficient to diminish NF-κB activation and amoeboid features. An FDA-approved antagonist of IL-1R for rheumatoid arthritis has been evaluated for the clinical management of cancer metastasis (Holen et al., 2016) (ClinicalTrials.gov identifier: NCT00072111) and a monoclonal antibody against IL-1α (Hong et al., 2014) is in phase III of clinical trials for colorectal cancer (ClinicalTrials.gov identifier: NCT01767857).

In conclusion, regional distribution of amoeboid melanoma cells with high Myosin II activity can contribute to lack of therapy responses by establishing a tumor-supportive AATME. We propose that, after surgical removal of the primary melanoma lesion, the amoeboid phenotype should be targeted using either ROCKi or IL-1α blocking antibodies as therapies to restrict immunosuppressive microenvironments and metastatic dissemination.

## STAR★Methods

### Key Resources Table

**Table:** 

REAGENT or RESOURCE	SOURCE	IDENTIFIER
**Antibodies**		

CD14- PerCP-Cy5.5 (clone HCD14)	Biolegend (for FC)	Cat# 325621; RRID:AB_893252
CD163 (clone EDHu-1)	AbD Serotec (for IHC)	Cat# MCA1853; RRID:AB_2074540
CD163-APC ((clone: eBioGHI/61 (Eghi/61))	eBioscience (for FC)	Cat# 17-1639-41; RRID:AB_2573167
CD206	Abcam (for IHC)	Cat# ab64693; RRID:AB_1523910
CD206-PE (clone 15-2)	Biolegend (for FC)	Cat# 321105; RRID:AB_571910
CD31	Abcam (for IHC)	Cat# ab28364; RRID:AB_726362
CD68 /pre-diluted (clone: KP-1)	Abcam (for IHC)	Cat# ab955; RRID:AB_307338
CD86-PeCy7 (clone IT2.2)	eBioscience (for FC)	Cat# 305421; RRID:AB_2275754
Cofilin (clone D59)	Cell Signaling Technology (for WB)	Cat# 3318; RRID:AB_2080595
CXCL8/IL8 (clone 6217)	R&D Systems (Neutralization)	Cat# MAB208; RRID:AB_2249110
F4/80	Abcam (for IHC)	Cat# ab100790; RRID:AB_10675322
GAPDH (clone 6C5)	Milipore (for WB)	Cat# MAB374; RRID:AB_2107445
GFP	ThermoFischer Scientific (for IHC)	Cat# A11122; RRID:AB_221569
HLA-DR- FITC (clone L243)	eBioscience (for FC)	Cat# 11-9952-41; RRID:AB_2572541
IgG1(clone 11711)	R&D Systems (Neutralization)	Cat# MAB002; RRID:AB_357344
IgG2α (clone 20102)	R&D Systems (Neutralization)	Cat# MAB003; RRID:AB_357345
IL1α/IL1F1 (clone 4414)	R&D Systems (Neutralization)	Cat# MAB200; RRID:AB_2295862
IκBα	BD Biosciences (for WB)	Cat# 610690; RRID:AB_398013
MLC2	Cell Signaling Technology (for WB)	Cat# 3672; RRID:AB_330278
NF-κB (p65)	Cell Signaling Technology (for IF)	Cat# 4764; RRID:AB_823578
Phospho-cofilin (Ser3)	Cell Signaling Technology (for WB)	Cat# 3311; RRID:AB_330238
Phospho-IκBα (Ser 32) (clone 14D4)	Cell Signaling Technology (for WB)	Cat# 2859; RRID:AB_561111
Phospho-MLC2 (Ser19)	Cell Signaling Technology (for IF, IHC)	Cat# 3671; RRID:AB_330248
Phospho–MLC2 (Thr18/Ser19)	Cell Signaling Technology (for WB)	Cat# 3674; RRID:AB_2147464
ROCK1	BD Biosciences (for WB)	Cat# 611137; RRID:AB_398448
ROCK2	BD Biosciences (for WB)	Cat# 610623; RRID:AB_397955
S100 beta	Abcam (for IHC)	Cat# ab52642; RRID:AB_882426
VE-cadherin	BD Transduction Laboratories (for IF)	Cat# 610252; RRID:AB_2276073

**Biological samples**		

Melanoma Tissue Microarrays (cohort A)	Hospital Universitari Arnau de Vilanova in Lleida, Spain	N/A
Melanoma tissues (cohort B)	King’s College London and National Institute for Health Research Biomedical Research Centre at Guy’s and St Thomas’ Hospitals	N/A
Sera samples from healthy donors and patients with melanoma	King’s College London and National Institute for Health Research Biomedical Research Centre at Guy’s and St Thomas’ Hospitals	N/A

**Chemicals, Inhibitors and recombinant proteins**		

IL4	Peprotech	Cat#200-04
IL10	Peprotech	Cat#200-10
IFNγ	Peprotech	Cat# 300-02
LPS	Sigma	Cat#0111:B4
M-CSF	Peprotech	Cat#300-25
H1152	Calbiochem	Cat#555550
GSK269962A	Axon Medchem	Cat#Axon 1167
Y27632	Tocris Bioscience	Cat#1254
(±) - Blebbistatin	Merck Calbiochem	Cat#203390
Brefeldin A *Biochemica*	AppliChem	Cat#A2138
LIMKi 3	Tocris Bioscience	Cat#4745
IKKβ Inhibitor III, BMS-345541	Merck	Cat#401480
Fluorescein isothiocyanate (FITC)–dextran	Sigma Aldrich	Cat#46944
CFSE Cell Division Tracker Kit	Biolegend	Cat#423801
DAPI (4’,6-Diamidino-2-Phenylindole, Dilactate)	Biolegend	Cat#422801
Clodronate liposomes and control liposomes	Liposoma B.V.	Cat#CP-025-025

**Experimental Models: Cell lines**		

A375P	Prof. Richard Hynes HHMI, MIT, US	ATCC CRL-1619
A375M2	Prof. Richard Hynes HHMI, MIT, US	Clark et al., 2000
WM88	Wistar Collection at Coriell Cell Repository	WC00123
WM3854	Wistar Collection at Coriell Cell Repository	WC00125
WM983A	Wistar Collection at Coriell Cell Repository	WC00048
WM983B	Wistar Collection at Coriell Cell Repository	WC00066
WM793B	Wistar Collection at Coriell Cell Repository	WC00062
WM1366	Prof Richard Marais Cancer Research UK Manchester Institute	Rockland WM1366-01-0001
HMVEC-dAd – Human Dermal Microvascular Endothelial Cells – Adult	Lonza	CC-2543
HUVEC – Human Umbilical Vein Endothelial Cells, Pooled, in EGM™-2	Prof A. Ridley University of Bristol, UK (original source: Lonza)	C2519A
U-937	Dr S. Karagiannis (KCL) (original source: ATCC)	ATCC® TIB-202
THP-1	Dr S. Karagiannis (KCL) (original source: ATCC)	ATCC® CRL-1593.2
5555	Prof Richard Marais Cancer Research UK Manchester Institute	Dhomen et al., 2009
B16F10	Prof Benilde Jimenez UAM-CSIC, Spain	ATCC CRL-6475

**Experimental Models: Organisms**		

SCID; CB17/Icr-Prkdcscid/IcrIcoCrl 5-6 weeks old female (for clodronate *in vivo* and A375P/M2 and WM983A/B xenografts)	Charles River	N/A
C57BL/6J 6-10 weeks old female (for 5555 *in vivo*)	The Jackson Laboratory	N/A
C57BL/6J 5-6 weeks old female (for B16F10 *in vivo*)	Charles River	N/A
NOD SCID gamma (NSG) NOD.Cg-*Prkdc*^*scid*^*Il2rg*^*tm1Wjl*^/SzJ 6-12 weeks old male and female- age- and sex-matched between groups (for dextran *in vivo* vascular permeability)	Charles River	N/A

**Oligonucleotides**		

Human NFKB1	Thermo Fisher Scientific	
siGENOME SMARTpool (#1)		D-003520-01
GCAGGUAUUUGACAUAUUA		D-003520-02
GCAAUAGCCUGCCAUGUUU		D-003520-03
GAACCACGCCUCUAGAUAU		D-003520-05
GGGCUACACCGAAGCAAUU		J-003520-07; #2
ON-TARGET plus		J-003520-08; #3
GAUGGGAUCUGCACUGUAA		
NF-κB1, ON-TARGET plus		
GAAAUUAGGUCUGGGGAUA		
Human ROCK1 ON-TARGET plus CCAGGAAGGU AUAUGCUAU	Dharmacon	J-003536-08-0005
Human ROCK2 ON-TARGET plus GAAACUAAUA GGACACUAA	Dharmacon	J-004610-08-0005
Human MYL12B	Dharmacon	
ON-TARGET plus SMARTpool		L-018116-01-0005
CCACUUAGCACUUGUAUAA		J-018116-09
GGGUGUAAAUUGUAUUGAA		J-018116-10
CCUCAUAGAACCUGUUGCA		J-018116-11
UGUAUUUAUUCCAGACCUU		J-018116-12
Non-targeting siRNA	Dharmacon	
ON-TARGET plus		D-001810-01-050
UGGUUUACAUGUCGACUAA		

**Software**		

GraphPad Prism	GraphPad Software, San Diego USA	Version 6
MetaCore ©	Thomson Reuters	
ImageJ	NIH	N/A
FlowJo	LLC	Version 7.6.5

**Molecular probes**		

Alexa Fluor 488 Phalloidin	ThermoFischer Scientific (for IF)	Cat# A-12379; RRID:AB_2315147
Hoechst	Invitrogen (for IF)	Cat# H1399

### Contact and Reagent Resource Sharing

Further information and requests for resources and reagents should be directed to and will be fulfilled by the Lead Contact, Victoria Sanz-Moreno (**v.sanz-moreno@qmul.ac.uk****)**

### Human sample collection and patient information

Patients were staged and classified according to the American Joint Committee on Cancer Melanoma Staging and Classification criteria (Balch et al., 2009). Human samples were collected with informed written consent, in accordance with the Helsinki Declaration, and the study design was approved by the Guy’s Research Ethics Committee and Ethics Committee of Guy’s and St Thomas’ NHS Foundation Trust and the Ethics Committee of the IRBLleida Biobanc, in accordance with the Human Tissue Act, 2004. This study was approved by the Guy’s Research Ethics Committee, study number 08/H0804/139. Tables S2–S4 show clinical information from human melanoma patients.

#### Cell culture

A375P, A375M2, WM88, WM1366, 5555 and B16F10 cells were maintained in DMEM, containing pyruvate, 4.5 g/ml D-Glucose, supplemented with 10% fetal calf serum (FCS), 100 units/ml penicillin, 100 μg/ml streptomycin and 2 mM L-glutamine and incubated at 37°C, 10% CO_2_. WM983A, WM983B, WM783B and WM8354 cells were cultured in RPMI supplemented with 10% FCS, 2mM L-glutamine, 100 units/ml penicillin and 100 μg/ml streptomycin and incubated at 37°C, 10% CO_2_. HUVECs were cultured in EBM2 media containing 2% fetal bovine serum (FBS), 0.1% hydrocortisone, 0.4% hFGF-b, 0.1% VEGF, 0.1% R3-IGF, 0.1% ascorbic acid, 0.1% hEGF, 0.1% GA-1000 and 0.1% heparin and cultivated on fibronectin coated flasks. HMVECs from a single neonatal donor were cultured in EGM-2MV bullet kit media comprised of EBM-2 Basal Medium, 5% FBS and EGM-2 MV growth supplements and were cultivated on gelatin-coated flasks. Both HUVEC and HMVEC were maintained in a 37°C, 5% CO_2_ incubator. THP-1 and U937 were cultured in RPMI supplemented with 10% FCS, 2mM L-glutamine, 100 units/ml penicillin and 100 μg/ml streptomycin and incubated at 37°C, 5% CO_2_. Primary monocytes were cultured in RPMI supplemented with 10% FCS, 2mM L-glutamine, 100 units/ml penicillin and 100 μg/ml streptomycin and incubated at 37°C, 5% CO_2_.

#### Cell culture on thick layers of collagen I

Fibrillar bovine dermal collagen (no. 5005-B; PureCol, Advanced BioMatrix) was prepared at 1.7 mg/ml in DMEM; 100 μl/well in 96-well plates; 700 μl/well in 12-well plates. After collagen gel polymerization (4 h), cells were seeded on top of collagen in medium containing 10% FCS, allowed to adhere for 24 h, treatments added (where appropriate), imaged and fixed or lysates collected. All assays were performed with melanoma cells seeded on top of a thick layer of collagen unless otherwise mentioned.

#### Generation of EGFP-tagged cell lines

pLNT/SFFV EGFP lentivector (1 μg) was transfected into HEK293T along with packaging vectors (p-MD2.VSVg (0.4 μg) and pΔ8.91 (1 μg)) using Lipofectamine 2000 (7.5 μl/well of a 6-well dish). Media was replaced 6 h after transfection. Media with lentiviruses were collected 48 h after transfection, spun down, filtered (0.45 μm) and added to recipient cell lines (A375P, A375M2, WM983A, WM983B and B16F10). EGFP-positive cells were FACS-sorted and used for subsequent experiments.

#### Generation of VENUS-5555 cells

HEK293T cells on 10 cm^2^ dishes were transfected using 2M CaCl_2_, and 2x HBS (51558, Sigma) with lentiviral Venus vector (15 μg), pMD2-VSVg (6 μg), pRSVrev (6 μg) and pMDL-g/p-RRE (6 μg). Supernatants were collected 48 h and 72 h after transfection. For lentiviral transduction, 10^5^ 5555 cells/well were seeded in 6-well tissue culture dish and infected with VENUS reporter lentiviruses added in suspension using 10 μg/ml Polybrene (107689, Sigma). After 48 h, successfully transduced cells were trypsinised and FACS sorted according to their VENUS expression.

#### ROCK inhibition and IKKβ inhibition

For ROCK inhibition, 1%-FCS media with inhibitors (5 μM H1152 or 10 μM Y27632) was added to cells for 4, 24 or 48 h with re-addition at 24 h. For IKKβ inhibition, IKKβ inhibitor III (0.5 μM) was used for 24 h. DMSO was used a vehicle in the same concentrations as the inhibitors. For the time-course experiments, the inhibitors were used at the same concentration for all the indicated time-points.

#### Melanoma secreted media (conditioned media, CM)

A375P, A375M2, WM983A, WM983B, WM88 and WM793B (2.5x10^5^ cells/well) were seeded in complete DMEM or complete RPMI (+10% FCS) in 6-well plates. Next day cells were washed with PBS (with calcium and magnesium) and were cultured in serum-free (SF) media for 48 h. For inhibitor treatments, H1152 (5 μM), Y27632 (5 μM) or GSK269962A (5 μM) was added in serum-free media and replenished after 24 h. Then media was collected, spin down to eliminate debris and used fresh in subsequent experiments. Recipient cells on collagen matrices were treated with secreted media for 24 h and then cell morphology and p-MLC2 levels were assessed. For the experiments with CM derived from melanoma cells isolated from TB or IF of A375M2 tumors (see also Diagram 1 in Methods S1), CM was generated similarly to above. For experiments using blocking antibodies, A375M2-derived media was pre-incubated with blocking antibodies (anti-IL1α, anti-IL8 and their respective isotype controls (IgG2A and IgG1), 0.75μg/ml) for 1 h at 37°C. Then, media was added to A375P cells seeded on coverslips (1 h treatment, for p65 immunofluorescence) or to A375P cells seeded on top of collagen (24 h treatment, for cell morphology analysis). For macrophage polarization experiments, the secreted media was concentrated using Amicon Ultra-4 Centrifugal Filter Unit with Ultracel-3 membrane (Millipore, Watford, UK). After centrifugation for 45 min at 4000 rpm at 4°C, protein concentration was measured by BCA Protein Assay Kit (Life Technologies).

#### Transfection and RNAi

2x10^5^ melanoma cells/well were seeded on 6-well plates and transfected the next day with 20-40 nM SmartPool or individual OTs (On Target) siRNA oligonucleotides, using Optimem-I and Lipofectamine 2000 (Invitrogen). Non-targeting siRNA was used as control. In case of transfection for siRNA to MYL12B and ROCK1/2, transfected cells were incubated for 24 h and 48 h, respectively, after which they were harvested and re-seeded at 25x10^4^ cells/well for conditioned media experiments and serum starved for 48 h.

#### Human PBMC isolation

Peripheral blood mononuclear cells (PBMC) from healthy donors were obtained from anonymized human buffy coats supplied by the NHS Blood and Transplant (Tooting, London, UK). Buffy coat was diluted with PBS (GIBCO) and PBMC isolation was performed by Lymphoprep density gradient separation (Axis-Shield, Oslo, Norway). MACS technology was used to isolate CD14+ monocytes.

#### Human CD14+ monocytes isolation

##### *In vitro* differentiation of human CD14+ monocytes to macrophages

For melanoma conditioned macrophage differentiation, 10^6^ CD14+ monocytes per well (6-well dish) were seeded in complete RPMI and incubated in 5% CO_2_ at 37°C. On day 3, 50% of the media was replenished with fresh media containing 35 μg/ml of CM derived from A375P, A375M2, H1152- or GSK269962A-treated A375M2 melanoma cells or CM derived from A375M2 depleted from MLC2 (MYL12B) or NFKB1 and monocytes were incubated for 3 additional days. A panel of known stimuli was used as controls of different types of differentiated/polarized macrophages. M-CSF (50 ng/ml), IL-4 (20 ng/ml) or IL-10 (20 ng/ml), or IFN-γ (20 ng/ml) plus LPS (100 ng/ml) were added on day 3 to monocytes after media replenishment. CD14+ cells with media only served as control. All cytokines were from Peprotech (London, UK) while LPS (derived from *Escherichia coli* 0111:B4) was from Sigma (Dorset, UK).

For macrophage differentiation with melanoma patient-derived serum (MPS), CD14+ monocytes (10^6^/ml) from healthy PBMC were plated in SF-RPMI and 10% of melanoma patient serum was added per well. Serum from either healthy volunteers (HVS) or human AB pooled serum (Sigma, Dorset, UK), were used as controls in the same volumes. Cells were incubated for 6 days in 5% CO_2_ at 37°C. HVS samples were obtained from King’s College London and National Institute for Health Research Biomedical Research Centre at Guy’s and St Thomas’ Hospitals. All sera samples used in these experiments were allogeneic to CD14+ monocytes. See also Table S2 for clinical information.

On day 6, cell supernatants were collected for the detection of cytokines. PBS −/− was added on cells and plates were placed on ice for 20-30 min. Cells were then scrapped using a p1000 pipette and used for phenotypic staining.

#### Macrophage morphology quantification

Bright-field images from day 6 of cell culture (see also ‘*In vitro* differentiation of human CD14+ monocytes to macrophages’ section) were used to analyze macrophage morphology using ImageJ software. Cells were divided into three categories according to their shape: rounded-immature, ‘fried-egg’ and spindle (Eligini et al., 2013) and one field of view was evaluated per condition. Data from 3 independent experiments with cells from 3 different healthy donors were used.

#### Zymosan phagocytosis assay *in vitro*

Zymosan A *S. cerevisiae* fluorescein-conjugated BioParticles (Life Technologies, Paisley, UK) were sonicated (3x20s; 90 ultrasonic watts) to obtain a homogeneous population. Zymosan particles were re-suspended in PBS−/− (10^8^ particles/ml) and opsonized with an equal volume of human AB pooled serum (Sigma) for 1h at 37°C. Differentiated macrophages were serum-starved for 2 h before starting the assay. Particles were thoroughly washed and added in a ratio of 10:1 (particles:macrophages) to macrophages and incubated for 1 h at 4°C to allow particles to bind on the cells and synchronize the onset of phagocytosis. Then cells were washed to remove unbound particles and incubated for 1 h at 37°C, 5%CO_2_, after which cells were harvested with PBS−/−, washed with FACS buffer and fixed in 1% para-formaldehyde to quench phagocytosis. Cells were acquired on a BD FACS CANTO II. As a negative control of phagocytosis, macrophages were treated with 5 μM cytochalasin D (Insight Biotechnology, Wembley UK), which is an inhibitor of actin polymerization-dependent phagocytosis. The inhibitor was added to the relevant wells 1 h before adding the zymosan particles and maintained throughout the assay.

#### Macrophage cytotoxicity/tumor cell killing assay *in vitro*

Briefly, melanoma target cells (A375M2, WM88, WM1366, WM793B, WM3854 and WM983A) were trypsinized, counted and labeled with CFSE (5 μM) using the CFSE cell division tracker unit from Biolegend following manufacturer’s instructions. Both CFSE-labeled melanoma cells and macrophages were washed twice in complete RPMI before the co-culture. Targets (10^4^ melanoma cells) were added on macrophages (10^5^ macrophages) in a ratio of 1:10 (melanoma targets:macrophages) in complete RPMI and incubated for 48 h in 48-well plates in duplicates. Co-cultured cells were harvested with PBS −/− and washed with FACS buffer. Cells were re-suspended in DAPI solution (5 μg/ml) for viability staining and immediately acquired on a BD FACS CANTO II.

#### Tumor cell isolation from tumor body and IF

Tumors were dissected from mice, transferred in falcon tubes with PBS −/− CaCl_2_/ MgCl_2_ and kept in ice before subjecting to tumor cell isolation. Tumors were sliced in the middle into two parts using a scalpel; one part was kept in 4% formaldehyde solution for 48 h for paraffinization while the second part was used for tumor cell isolation. For the latter, the tumor half was peeled with the use of forceps, a pair scissors and scalpel to collect the peritumoral area which was more transparent, while the tumor core was a solid, darker area. Tumor core and peritumoral area were both collected and chopped into small pieces and incubated with digestion solution (1ml/sample); (Digestion solution: 90μl Liberase TM (Roche), (5 mg/ml), 90μl Liberase TH (Roche), (5 mg/ml), 30μl DNase I (Sigma), (5 mg/ml) and 6 mL HBSS (GIBCO)) at 37°C with shaking. See also Diagram 1 in Methods S1.

#### Chemotaxis assay

Chemotaxis of human primary peripheral blood monocytes, THP1, U937 was assessed using 6.5-mm Costar Transwell cell culture chamber with polycarbonate membrane (5.0 μm pore) following the manufacturer’s protocol. Cell suspensions of 3x10^5^ cells/0.1ml in SF-RPMI were loaded in the upper chamber compartment. CM from A375M2 or A375P were loaded in the lower chamber. Migrated cells were counted using a Neubauer Chamber. For chemotaxis of A375M2 or A375P cells, 8.0 μm-pore transwells were used instead, and the cell suspension was of 2x10^5^ cells/0.1ml in SF-DMEM.

#### Transendothelial permeability assay

CM from A375P, A375M2 control, or ROCK1/2 or NFKB1-depleted A375M2, or A375M2 cells treated with ROCK inhibitors H1152 (5 μM) or Y27632 (10 μM) for 48 h were collected and added to confluent monolayers of HMVECs on gelatin-coated Transwell filters (Costar). FITC-dextran (0.1 mg/ml) was added to the top chamber. Samples from the lower chamber were removed after 1 h incubation and added to a black 96-well plate. Fluorescence was measured using a microplate analyzer. Each condition was performed in triplicates.

#### Endothelial cell proliferation *in vitro*

HMVECs or HUVECs cells were seeded in 6 replicates (10^4^/well) on 1% gelatin (Sigma, Dorset, UK)-coated 96-well plates in complete EGM-2MV medium. The following day, cells were washed with PBS (+/+) and treated with 50% supernatant derived from monocytes treated with melanoma-derived CM (A375M2 +H1152 or GSK269962A), from untreated monocytes or with EGM-2MV medium (as control) for 72 h. Proliferation was measured by an MTT assay (5 mg/ml; Sigma, Dorset, UK). The absorbance of the samples at 570 nm and 695 nm was measured on a Perkin Elmer/Packard fusion Alpha-FP microplate analyzer. The results are presented as the average values of 6 replicates after background subtraction (O.D. 570 nm - O.D. 695nm).

#### Human cytokine array

Secreted media from A375M2 and A375P cells seeded on a thick layer of collagen I were collected after 48 h and incubated with Human Cytokine Antibody Array (RayBiotech, Inc., C4000) following the manufacturer’s protocol. Membranes were incubated with biotinylated detection antibody cocktail, with HRP-conjugated streptavidin and with detection buffers. Images were obtained with a chemoluminiscent imaging system and densitometry analysis was performed using the Protein Array Analyzer plugin for ImageJ (http://image.bio.methods.free.fr/ImageJ/?Protein-Array-Analyzer-for-ImageJ.html). Enrichment maps and networks and process networks were obtained using MetaCore software from Thomson Reuters (https://thomsonreuters.com/metacore/).

#### Human cytoskeleton phospho-array

HMVECs were incubated with CM derived from A375M2 or ROCK1/2-depleted A375M2 (48 h) and after 1 h, cells were lysed using a protein extraction buffer and lysis beads from the Cytoskeleton Phospho Antibody Array (Full Moon Biosystems, CP141). Extracted protein was purified through columns and quantified measuring UV absorption. Protein lysate (40 μg) was biotinylated and conjugated to the antibody array. This array contains antibodies against 141 proteins involved in cytoskeletal pathways.

#### Immunoblotting

Cells were lysed in Laemmli Buffer and lysates were resolved by 10 or 12% SDS-polyacrylamide (PAGE) gels or pre-made Nu-PAGETM 4%–12% Bis-Tris gels (Invitrogen) and transferred to PVDF filters (0.45 μm, Immobilon). The ECL Plus or Prime ECL detection systems (GE Healthcare) with HRP-conjugated secondary antibodies (GE Healthcare) were used for detection. Bands were quantified using ImageJ (https://imagej.nih.gov/ij/).

#### ELISA experiments

CM from melanoma cells or supernatants from macrophages were used for sandwich ELISA experiments using commercially available kits (see also the table for reagents above). 96-well NUNC clear flat-bottom plates were coated with antibodies (IL-10, IL-8, IL-1α and TNF-α) and incubated O/N at 4°C in a humified chamber. ELISA MAX™ standard sets (Biolegend) were used for the detection of IL-10, IL-8 and TNF-α; ELISA MAX Deluxe (Biolegend) for IL1-α; and for TGF-β, LEGEND MAX Total TGF-β1 ELISA Kit with pre-coated plates was used (Biolegend). Microwell absorbance was read at 450 nm on a Thermo Scientific Multiskan EX microplate reader. All samples were quantified based on a standard curve using Microsoft Excel.

#### Immunofluorescence

HMVECs seeded onto gelatin-coated coverslips were incubated in the presence of media derived from A375M2 cells, ROCK-depleted A375M2 cells or ROCK-inhibited A375M2 cells for 1 h. Cells were fixed with 4% p-formaldehyde, permeabilised with 0.3% Triton and stained with phalloidin for F-actin, with anti-VE-cadherin and with Hoechst 33258 for the nuclei. A375M2 or A375P cells were seeded on top of collagen gels and treated as indicated. Cells were fixed, permeabilized, incubated with primary antibody (pMLC Ser19, Cell Signaling) and stained with secondary Alexa Fluor-647 anti-rabbit (Life Technologies) and Alexa Fluor 546-phalloidin for F-actin detection (Life Technologies). For imaging, gels were inverted onto MatTek dishes, while coverslips were mounted with DAKO fluorescence mounting medium (Dako, Cambridgeshire). In both cases, images were taken with a Zeiss LSM 510 Meta confocal microscope (Carl Zeiss, Germany) with C-Apochromat 40 × /1.2 NA (water) objective lens and Zen software (Carl Zeiss).

For cell morphology, the shape descriptor “roundness” in ImageJ was used after manually drawing around the cell shape using F-actin staining images (Orgaz et al., 2014). Phospho-MLC2 fluorescence signal was quantified calculating the pixel intensity in single cells relative to the cell area (Orgaz et al., 2014).

Junctional index and gap area parameters were measured with ImageJ. Junctional index is calculated as described by Cain et al. (2010).

For p65 staining, cells seeded on glass coverslips were fixed with 4% formaldehyde for 15 min and washed with PBS (3 × 5 min). Then, cells were permeabilised for 20 min with 0.5% Triton X-100 in 4% BSA-PBS and washed 3 times with PBS. After blocking for 30 min, cells were incubated with anti-p65 (1:100) overnight at 4°C. Cells were then washed 3 times with PBS and incubated with anti-Rabbit Alexa Fluor 488 (1:350) and Alexa Fluor 546-phalloidin (1:350) for 2 h at room temperature. Finally, cells were washed with PBS 4 × 5 min, incubated with 5 μg/ml Hoechst 33258 in the 4th wash, and mounted on a slide with Fluoroshield mounting media. Blocking and antibodies were prepared with 0.2% Triton X-100 in 4% BSA-PBS.

#### Flow cytometry

Cells were washed once with FACS buffer (PBS−/−, 1% BSA, 2 mM EDTA, 0.1% NaN_3_) and after FcR blocking (Human TruStain FcX, Biolegend) co-stained for: HLA-DR-FITC (1:50, clone: L243), CD86-PE-Cy7 (1:20, clone: IT2.2), CD163-APC (1:20, clone: GHI/61); all from eBiosciences (Hertfordshire, UK) and CD206-PE (1:20, clone: 15-2), (Biolegend, London, UK). After 30 min incubation at 4°C in the dark, cells were washed twice with FACS buffer (1500 rpm, 4°C, 5 min) and re-suspended in 500 μl DAPI solution (5ug/ml, Biolegend) for viability and immediately acquired on a BD FACS CANTO II flow cytometer and analyzed using FlowJo 7.6.5 software (Tree Star). Purity of isolated CD14+ cells was checked by staining for CD14-PerCP-Cy5.5 (1:50, clone: HCD14, Biolegend) and was routinely > 95% across all the experiments.

#### Analysis of cytokine expression from human databases

Gene expression data of human melanoma samples from published microarray studies was used to analyze most of the cytokines enriched in media derived from A375M2 cells in melanoma progression. We only took into account studies with enough sample purity (> 95% melanocytic cells), enough patient samples to perform statistical comparisons (n > 40) and studies including normal tissue. From public database GEO we extracted the Avery (GEO Accession number GSE29359)(Avery-Kiejda et al., 2011), Xu (GEO Accession number GSE8401)(Xu et al., 2008), Talantov (GEO Accession number GSE3189)(Talantov et al., 2005), Kabbarah (GEO Accession number GSE46517)(Kabbarah et al., 2010) and Riker (GEO Accession number GSE7553)(Riker et al., 2008) series. Samples from these studies were reported to have > 95% melanocytic/melanoma cells and no mixed histology. Data were normalized using Gene Pattern (https://www.broadinstitute.org/cancer/software/genepattern/) and analyzed as described in the ‘Statistical analysis’ section below.

Gene expression data of human melanoma samples from The Cancer Genome Atlas (TCGA) database (https://cancergenome.nih.gov/) was also used to analyze cytokines (enriched in media from highly contractile A375M2 cells) expression in melanoma progression. We only took account patients who had not received neo-adjuvant treatment prior to the resection of the tumor that yielded the sample submitted for TCGA. Normalized expression data and z-scores for mRNA expression data were donwloaded from cBio-Portal.

#### Animal welfare

All animals were maintained under specific pathogen-free conditions and handled in accordance with the Institutional Committees on Animal Welfare of the UK Home Office (The Home Office Animals Scientific Procedures Act, 1986). All animal experiments were approved by the Ethical Review Process Committee at King’s College London and carried out under license from the Home Office, UK.

#### Melanoma tumor models

All animal procedures were approved and carried out in accordance with the UK Home Office and an Ethical Review Panel. All mice were obtained from Charles River. Severe combined immunodeficient mice (SCID; CB17/Icr-*Prkdc^scid^*/IcrIcoCrl) were used for studies with human melanoma cell lines (for A375P-, A375M2-, WM983A- and WM983B-EGFP cell lines) while C57BL/6J mice were used with mouse melanoma cell lines (B16F10-EGFP and 5555-Venus). Mice were female 5-6 weeks old for all experiments except for experiments using 5555 cells (6-10 weeks old).

Prior to injection, cells were counted and resuspended in PBS −/− CaCl_2_/ MgCl_2_. Mice were anaesthetized with isoflurane and cells (2 × 10^6^ for A375P, A375M2 and WM983A, WM983B; and 1.5 × 10^5^ for B16F10) in a volume of 50 μL were subcutaneously injected on the flank. Mice were continually monitored with tumor dimensions being determined by calliper measurements. Tumor volume (mm^3^) = length x width x height x 0.52.

Visualization of tumor fluorescence was achieved using a Fluorescent Protein Flashlight (NIGHTSEA) post injection and upon tumor establishment. A375s and WM983s tumors were grown for 33 and 55 days, respectively, while B16F10 tumors were grown for 15 days. Prior to dissection all tumors were measured, dissected out, surrounding tissue removed, followed by fixation in 4% formaldehyde solution for 48 h. All dissected tumors were weighed, and fluorescence images taken using a Fluorescence labeled Organism Bioimaging Instrument (FOBI; NeoScience).

For Y27632 ROCK inhibitor studies, drug administration was by intraperitoneal injection every other day once tumors had reached a mean volume of 160 mm^3^. Dosing was at 50 mg/kg (Y27632; Bio-Techne). Y27632 was prepared in PBS −/− CaCl_2_/ MgCl_2_, and sterile filtered prior to use.

For experiments using 5555 cells, prior to injection 5555-Venus cells were pre-treated *in vitro* with GSK269962A (5 μM) or DMSO as control for 5 days, with fresh drug added every day. Then 2x10^5^ cells were injected intradermally into C57BL/6J mice. Both *in vitro* and *in vivo* viability was measured after drug removal. Therefore, mice were never treated during the course of the *in vivo* experiment. Tumor volume was measured twice a week and tumors harvested after 8 and 14 days for further analyses.

#### Dextran *in vivo* vascular permeability

A375M2 cells were transfected with either control siRNA or siROCK1/2 (60 nM total for both). Three days later, cells were labeled with 10 μM CMFDA-Green (C7025, Life Technologies) for 10 min and then they were trypsinized and counted. 1x10^6^ labeled cells/0.1ml PBS were injected into tail vein of NOD/SCID/ IL2Rγ−/− mice (NSG, Charles River). 24 h later, TRITC-dextran (70 kDa, D1818, ThermoFisher) was intravenously injected and 10 min later mice were sacrificed. Lungs were extracted, washed with PBS (with calcium/magnesium) twice and fixed with 4% formaldehyde for 16 h at 4°C. Lungs were examined under a confocal microscope (see Immunofluorescence section). Data are presented as % field of area covered by fluorescence (green for cells, red for dextran which represents permeability), n = 5 mice/condition for each experiment, n = 2 independent experiments.

#### Macrophage depletion *in vivo* by clodronate administration

Severe combined immunodeficient mice (SCID; CB17/Icr-*Prkdc*^*scid*^/IcrIcoCrl; Charles River) were injected intraperitoneally (i.p.) with 150 μl of clodronate liposomes or PBS liposomes as control (cat# CP-025-025, Liposoma B.V. the Netherlands). Next day, tumor cells (2x10^6^ cell/ 0.1ml PBS) were injected subcutaneously along with 20 μl of clodronate or PBS liposomes into mice. Then, 150 μl clodronate or PBS liposomes was administered i.p. twice per week. Tumor volume was monitored every 3 days by caliper measurements as in “Melanoma tumour models” section. On day 27, mice were culled and tumors were harvested, fixed and embedded in paraffin using standard protocols (see “Immunohistochemistry” section). Macrophage depletion was confirmed by F4/80 and CD206 IHC staining at endpoint.

#### Immunohistochemistry

##### Case selection

Two cohorts of human melanoma samples were included in the case series. The ‘cohort A’ is a tissue microarray comprising of two slides with consecutive sections of individual tumor cores corresponding to either TB or IF for 40 different patients (n = 24 primary and n = 16 metastasis). Each patient is represented by 8 cores (4 replicates/tumor body and 4 replicates/IF) so 320 cores were totally analyzed for each marker. The ‘cohort B’ was comprised by whole-section tissues corresponding to 7 different patients.

Whole sections from subcutaneous tumors (human A375P-, A375M2-, WM983A- and WM983B-EGFP; and murine B16F10-EGFP) and from intradermal tumors (5555-Venus) were included.

##### Experimental procedure

All tissue samples were formalin-fixed paraffin-embedded (FFPE) and were sectioned (3 or 4 μm-thick) and dried for 1 h at 65°C. Next, tissue samples were subjected to deparaffinization, rehydration and heat-induced epitope retrieval using a Biocare Decloaking Chamber (DC2012) at 110°C for 6 min in Access Super Menarini Buffer (MP-606-PG1). Then endogenous peroxidase and phosphatase alkaline were blocked with Dual Endogenous Enzyme-Blocking Reagent (Dako, Agilent) for 10 min.

Incubation with primary antibodies was performed O/N at 4°C in a humidified slide chamber. Human samples were subjected to IHC for pSer19-MLC2 (1:50, polyclonal, #3671), CD68 (1:2 dilution/pre-diluted; clone: KP-1, #ab74704), CD163 (1:100, clone: EDHu-1, MCA1853), CD206 (1:2000, ab64693) and CD31 (1:100, ab28364). Murine tumor samples were stained for GFP (1/2000, A-11122), S100β (1/400, ab52642), F4/80 (1/400, ab100790), CD206 (1/2500, ab64693), CD31 (1/100, ab28364) and pSer19-MLC2 (1:50, polyclonal, #3671).

Relevant secondary antibodies IgG HRP (Horse-Radish Peroxidase) (1/100, anti-rabbit or anti-mouse, Dako) or AP (Alkaline Phosphatase) (1/100, anti-rabbit or anti-mouse, Dako) were incubated for 1 h at room temperature. Subsequently, samples were developed by incubation in DAB+ or Permanent Red chromogen solutions (Dako, Agilent). Samples were counter stained with hematoxylin. Human melanoma tissues and the adjacent normal skin were used as positive and negative controls for the above-mentioned antibodies.

##### Imaging and Scoring

Human tissue samples were imaged and scored as follows. Cohort A was scanned on a ZEISS Axio Scan.Z1, and images were analyzed by ZEN 2012 (blue edition). Cohort B was scanned using a Hamamatsu Nanozoomer and images analyzed using NDP view2. For the analysis, each individual tumor core was evaluated for the total number of vessels or macrophages and then averages of the numbers per IF or TB were calculated for each patient. For the comparison between primary and metastatic melanoma patients, averages from all the replicates (including TB and IF) were calculated. For vessel and macrophage analysis, ≥ 2 fields of view were used per TB or IF for analysis. The latter was defined as the tumor area composed by melanoma cells only with at least 50% cell surface in contact with the matrix (Cantelli et al., 2015, Sanz-Moreno et al., 2011).

Cell shape scoring was performed as previously described (Sanz-Moreno et al., 2011). Briefly, cell shape score = ((percentage of cells [%] shape 0 × 0) + (% shape 1 × 1) + (% shape 2 × 2) + (% shape 3 × 3)), with values ranging from 0 (all cells round) to 300 (all cells spindle). For quantification of p-MLC2 staining, samples were scored blind and a staining H-score was provided for each section, where H-score = Σ (% of cells with 4^∗^4) + (% of cells with 3^∗^3) +(% of cells with 2^∗^2) +(% of cells with 1^∗^1); where 0 = no staining, 1 = weak, 2 = moderate, 3 = intense and 4 = very intense staining.

For A375P/A375M2 and WM983A/WM983B tumors, samples were scored 0-4 using the H-score method for p-MLC2, in addition to quantification of the percentage of cells with very intense (H-score = 4) p-MLC2 levels at distances 0-2 mm from the IF of tumors.

For murine tumors, 6 consecutive sections from each sample were used. Two sections were used to detect F4/80+CD206+ macrophages, two to detect melanoma cells using the antigen S100β and GFP and two for p-MLC2 and CD31+. One area for IF and TB was selected for each sample and imaged at 20X magnification using iScope microscope (IS.1159EPLi, Euromex) and ImageFocus 4.0 (Euromex) software (Diagram 2A in Methods S1). Next, the images were run in ImageJ platform (https://imagej.nih.gov/ij/), scaled at 3.08 px/μm^2^. Color deconvolution H-DAB plug-in (https://imagej.net/Colour_Deconvolution) was applied to split the image in three main channels/colors. The area of interest was determined, and the number of positive cells was determined using “find maxima” tool. The process was iterated for all markers used in the analysis (F4/80, CD206, GFP and S100β) (Diagram 2B in Methods S1). All the values were introduced in an Excel file and were standardized to the same area (10^5^ μm^2^). To avoid false positive macrophages (that is cancer cells that were CD206+) we applied a correction factor: subtraction of GFP or S100β signal from CD206 total signal.

#### Statistical analysis

Statistical analysis was performed using GraphPad Prism (version 6, San Diego California USA). The following statistical tests were used: t test unpaired, t test with Welch’s correction, Mann-Whitney test, one-way ANOVA with Tukey post hoc, Kruskal-Wallis with Dunn’s multiple comparison test and Wilcoxon matched-pairs signed rank test and Two-way ANOVA- post with Bonferroni post hoc test. All experiments were analyzed with a minimum of three independent repeats. Outliers were excluded using the ROUT method. For column bar or scatter dot plots, error bars are the average ± SEM. Boxplots show min to max values or 10-90 percentile. ^∗^ p < 0.05, ^∗∗^ p < 0.01, ^∗∗∗^ p < 0.001, ^∗∗∗∗^ p < 0.0001.

## Supplemental Information

Document S1. Tables S1–S4

Methods S1. Diagrams S1 and S2
