# Supplementary material for: Regional Activation of Myosin II in Cancer Cells Drives Tumor Progression via a Secretory Cross-Talk with the Immune Microenvironment
Source: Cell. 2019 Feb 7;176(4):757–774.e23. doi: 10.1016/j.cell.2018.12.038 (PMC6370915; doi:10.1016/j.cell.2018.12.038)
Supplement: Document S1. Tables S1–S4 [file mmc1.pdf]

**Cell, Volume *176***

## Supplemental Information

# Regional Activation of Myosin II in Cancer Cells Drives Tumor Progression via a Secretory Cross-Talk with the Immune Microenvironment

**Mirella Georgouli, Cecilia Herraiz, Eva Crosas-Molist, Bruce Fanshawe, Oscar Maiques, Anna Perdrix, Pahini Pandya, Irene Rodriguez-Hernandez, Kristina M. Ilieva, Gaia Cantelli, Panagiotis Karagiannis, Silvia Mele, Hoyin Lam, Debra H. Josephs, Xavier Matias-Guiu, Rosa M. Marti, Frank O. Nestle, Jose L. Orgaz, Ilaria Malanchi, Gilbert O. Fruhwirth, Sophia N. Karagiannis, and Victoria Sanz-Moreno**

**SUPPLEMENTAL TABLES**

**Table S1: Chemokines that are up-regulated in CM from A375M2 cells vs. CM from A375P cells**

**Related to Figures 2 and 3**

| **Chemokines found up-regulated in CM A375M2 vs. CM A375P** |
|---|
| • MIP3alpha (CCL20)<br>• MIP1delta (CCL15)<br>• Fractalkine (CX3CL1)<br>• CCL14<br>• MCP4 (CCL13)<br>• Eotaxin (CCL11)<br>• MCP2 (CCL8)<br>• MIP1beta (CCL4)<br>• MIP3b (CCL19)<br>• IL8 (CXCL8)<br>• MIP1alpa (CCL3)<br>• MDC (CCL22)<br>• Eotaxin-2 (CCL24)<br>• CXCL16<br>• Resistin<br>• MPI1F1 (CCL23)<br>• BLC (CXCL13)<br>• GRO (CXCL1)<br>• DPPIV (CD26)<br>• TARC<br>• LAP |

**Table S2: Clinical information for sera samples obtained from melanoma patients**

**Related to Figure 3**

| **Patient** | **Stage of Melanoma** | **Gender** | **Age** | **Medication at sampling** | **Site of melanoma** |
|---|---|---|---|---|---|
| 1 | II | M | 75 | N/A | N/A |
| 2 | IV | F | 72 | N/A | Metastasis |
| 3 | IV | M | 44 | N/A | Metastasis |
| 4 | IV | M | 53 | Unknown | Unknown |
| 5 | IV | M | 26 | On Clinical trial, stable disease | Mid back |
| 6 | IV | M | 66 | Unknown | Unknown |
| 7 | IV | F | 41 | Unknown | Unknown primary site |
| 8 | IV | M | 81 | Maxillectomy, followed by adjuvant radiotherapy | left upper alveolus melanoma |
| 9 | IV | M | 67 | Unknown | Unknown |
| 10 | IV | F | 31 | Low dose Interferon | Left medial thigh |
| 11 | IV | F | 87 | Unknown | Left calf |
| 12 | IV | F | 61 | Unknown | Central forehead |
| 14 | II | M | 74 | None | Left shoulder |
| 15 | II | F | 63 | Unknown | Unknown |
| 16 | II | F | 54 | Unknown | Left knee |
| 17 | II | M | 38 | None | Left neck |
| 18 | II | M | 81 | Unknown | Unknown |
| 19 | II | M | 52 | Unknown | Right posterior thigh |
| 20 | II | F | 78 | Unknown | Right cheek |
| 21 | II | M | 76 | Simvastatin | Left upper back |
| 22 | II | M | 72 | Unknown | Left shoulder |
| 23 | II | F | 23 | Unknown | Right posterior auricular area |

**Table S3: Clinical information for melanoma patients of the cohort A**

**Related to Figure 1**

| Patient | Gender | Age | Classification |
|---|---|---|---|
| 1 | M | 86 | Primary |
| 2 | M | 58 | Primary |
| 3 | F | 86 | Primary |
| 4 | M | 70 | Primary |
| 5 | M | 83 | Primary |
| 6 | M | 89 | Primary |
| 7 | M | 70 | Primary |
| 8 | M | 61 | Primary |
| 9 | F | 65 | Primary |
| 10 | F | 62 | Primary |
| 11 | M | 64 | Primary |
| 12 | F | 85 | Primary |
| 13 | F | 59 | Primary |
| 14 | M | 62 | Primary |
| 15 | M | 82 | Primary |
| 16 | M | 91 | Primary |
| 17 | F | 80 | Primary |
| 18 | M | 86 | Primary |
| 19 | F | 75 | Primary |
| 20 | F | 76 | Primary |
| 21 | F | 32 | Primary |
| 22 | M | 67 | Primary |
| 23 | F | 32 | Metastasis |
| 24 | M | 74 | Primary |
| 25 | M | 65 | Metastasis |
| 26 | M | 65 | Metastasis |
| 27 | F | 87 | Metastasis |
| 28 | F | 42 | Metastasis |
| 29 | M | 59 | Metastasis |
| 30 | M | 52 | Metastasis |
| 31 | M | 69 | Metastasis |
| 32 | F | 84 | Metastasis |
| 33 | F | 52 | Metastasis |
| 34 | M | 72 | Metastasis |
| 35 | F | 72 | Metastasis |
| 36 | M | 58 | Metastasis |
| 37 | M | 70 | Metastasis |
| 38 | M | 82 | Metastasis |
| 39 | M | 65 | Metastasis |
| 40 | F | 71 | Metastasis |

**Table S4: Clinical information for melanoma patients of cohort B**

**Related to Figure 1**

| Patient | Gender | Age | Stage at sampling | Classification |
|---|---|---|---|---|
| 1 | M | 83 | IIC | Primary |
| 2 | N/A | N/A | N/A | Primary |
| 3 | N/A | N/A | N/A | Primary |
| 4 | F | 84 | IIIB | In transit metastasis |
| 5 | N/A | N/A | N/A | Lymph node metastasis |
| 6 | F | 92 | IIIB | In transit metastasis |
| 7 | M | 63 | IIIC | In transit metastasis |
