# Supplementary material for: Regional Activation of Myosin II in Cancer Cells Drives Tumor Progression via a Secretory Cross-Talk with the Immune Microenvironment
Source: Cell. 2019 Feb 7;176(4):757–774.e23. doi: 10.1016/j.cell.2018.12.038 (PMC6370915; doi:10.1016/j.cell.2018.12.038)
Supplement: Methods S1. Diagrams S1 and S2 [file mmc2.pdf]

Diagram 1

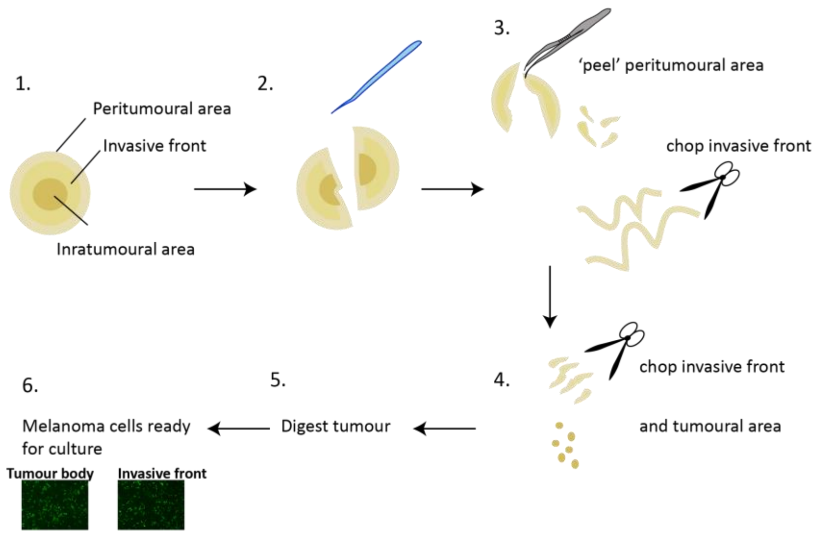

Diagram 2

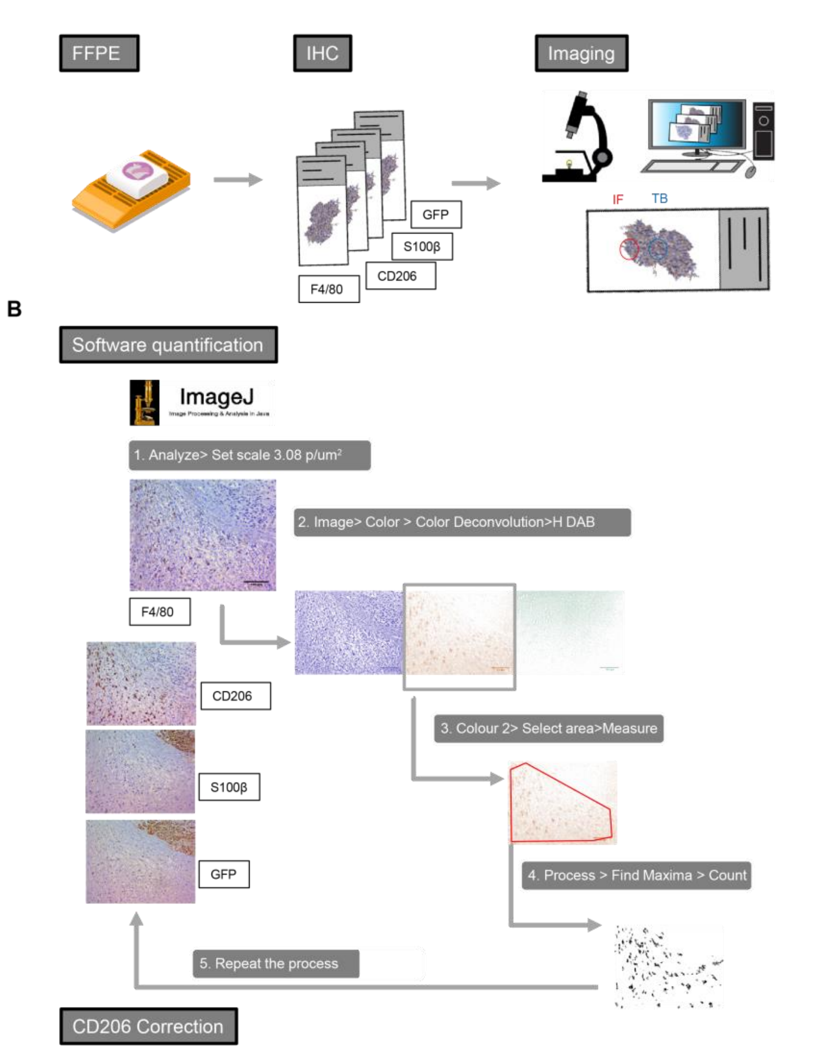

$$CD206_{corrected} = \frac{CD206_N - CancCells_N}{Area} \text{ x}10^5$$

* *Canc cells: Biggest value for either S100β or GFP staining*
